# Synthesis, Chemical–Physical Characterization, and Biomedical Applications of Functional Gold Nanoparticles: A Review

**DOI:** 10.3390/molecules26195823

**Published:** 2021-09-26

**Authors:** Ileana Ielo, Giulia Rando, Fausta Giacobello, Silvia Sfameni, Angela Castellano, Maurilio Galletta, Dario Drommi, Giuseppe Rosace, Maria Rosaria Plutino

**Affiliations:** 1 Institute for the Study of Nanostructured Materials, ISMN—CNR, Palermo, c/o Department of ChiBioFarAm, University of Messina, Viale F. Stagno d’Alcontres 31, Vill. S. Agata, 98166 Messina, Italy; ileana.ielo@ismn.cnr.it (I.I.); fausta.giacobello@ismn.cnr.it (F.G.); ssfameni@unime.it (S.S.); angela.castellano@ismn.cnr.it (A.C.); 2 Department of Chemical, Biological, Pharmaceutical and Analytical Sciences (ChiBioFarAm), University of Messina, Viale F. Stagno d’Alcontres 31, Vill. S. Agata, 98166 Messina, Italy; girando@unime.it (G.R.); mgalletta@unime.it (M.G.); ddrommi@unime.it (D.D.); 3 Department of Engineering, University of Messina, Contrada di Dio, S. Agata, 98166 Messina, Italy; 4 Department of Engineering and Applied Sciences, University of Bergamo, Viale Marconi 5, 24044 Dalmine, Italy

**Keywords:** gold nanoparticles, nanomaterials synthesis, drug delivery, photothermal activity

## Abstract

Relevant properties of gold nanoparticles, such as stability and biocompatibility, together with their peculiar optical and electronic behavior, make them excellent candidates for medical and biological applications. This review describes the different approaches to the synthesis, surface modification, and characterization of gold nanoparticles (AuNPs) related to increasing their stability and available features useful for employment as drug delivery systems or in hyperthermia and photothermal therapy. The synthetic methods reported span from the well-known Turkevich synthesis, reduction with NaBH_4_ with or without citrate, seeding growth, ascorbic acid-based, green synthesis, and Brust–Schiffrin methods. Furthermore, the nanosized functionalization of the AuNP surface brought about the formation of self-assembled monolayers through the employment of polymer coatings as capping agents covalently bonded to the nanoparticles. The most common chemical–physical characterization techniques to determine the size, shape and surface coverage of AuNPs are described underlining the structure–activity correlation in the frame of their applications in the biomedical and biotechnology sectors.

## 1. Introduction

Nowadays, nanotechnology and nanochemistry are very often combined in order to develop nanostructured materials and, also, determine to what extent the manipulation of matter on an atomic, molecular, and supramolecular level may affect the desired nanomaterials properties [1]. The atomic structure of materials having nanometric sizes promotes the implementation of their physical, chemical, and biological properties [2]. In particular, the electronic energy levels in nanomaterials are quantized and not continuous as to their corresponding bulk conformation; this effect, known as the quantum confinement effect, demonstrates that material properties are size-dependent [3]. The modification of surface area and electron delimitation, due to the confinement of electronic wave function in up to three physical dimensions, induces the development and the possibility to customize some properties, such as chemical reactivity, melting point, electrical conductivity, fluorescence, and magnetic permeability as a function of the size of nanoparticles [4]. The history of AuNPs dates back to remote times when red ruby glass began to be used; however, they received the maximum attention starting from the end of the seventeenth century. The properties of metallic gold, such as optical and thermal, are explained by describing the plasmon resonance, which makes the AuNPs employable as sensors [5], ultra-small light emitters [6], nano heaters [7], or nano antennas [8]. Gold is an element that has a singular mix of physical and chemical features both in macroscopic and microscopic conditions. While the macroscopic properties concern its unique yellow color, chemical stability, and high redox potential, at the nanometric level, gold features are explained by a combination of the electronic structure with other effects due to the extremely small dimensions. Moreover, this is also due to (i) a high ratio of surface atoms to bulk atoms, (ii) electromagnetic confinement due to a localized plasmon resonance after the interaction with an optical wave, and (iii) the quantum effects, which justify, for instance, the change from metallic to a semiconducting character [9]. One of the most impressive and useful AuNPs properties is plasmon resonance related to the collective behavior of conduction gold electrons. In fact, when it comes to metals, the conduction electrons behave as free charges, which can be excited by an electromagnetic wave. Thus, plasmon waves result from both charge mechanical oscillations and electromagnetic oscillations of the electric field. When this phenomenon occurs at the nanoscale, it is called Localized Surface Plasmon Resonance (LSPR), and it is the result of the confinement of the electric field within a small metallic sphere. This explains the red–purple color of spherical nanoparticles and its slight change when the shape or the surrounding medium are altered. For example, the LSPR is a powerful technique to input energy in metallic nanoparticles, enhancing the light-to-heat conversion. This study reports different chemical and green synthesis methods for the production of gold nanoparticles (AuNPs), namely, chemical reduction or others, such as electrochemical [10], thermal [11], and photochemical reduction techniques. The applications of AuNPs are strictly related to their shape and size; for example, gold nanorods are employed as biosensors, antineoplastic drugs [12], and as carriers in drug delivery systems [13]. AuNPs can penetrate cancer cell membranes, preventing their proliferation and growth [14]. When these nanoparticles interact with light, the oscillating electric field induces the conduction electrons to oscillate with the same frequency of the electromagnetic wave; this is coherent with their plasmon electron cloud and its distribution over the whole nanoparticle volume. The AuNP surface charge is neutralized through undesired aggregation phenomenon. This can be mitigated by using opportune functional capping agents and depositing them on the surface; they can be small molecules, polymers, or biomolecules [15]. Depending on these surface modifications, AuNPs can be employed in engineering, chemical, biochemical, and medical applications [16]. 

**Figure 1:**
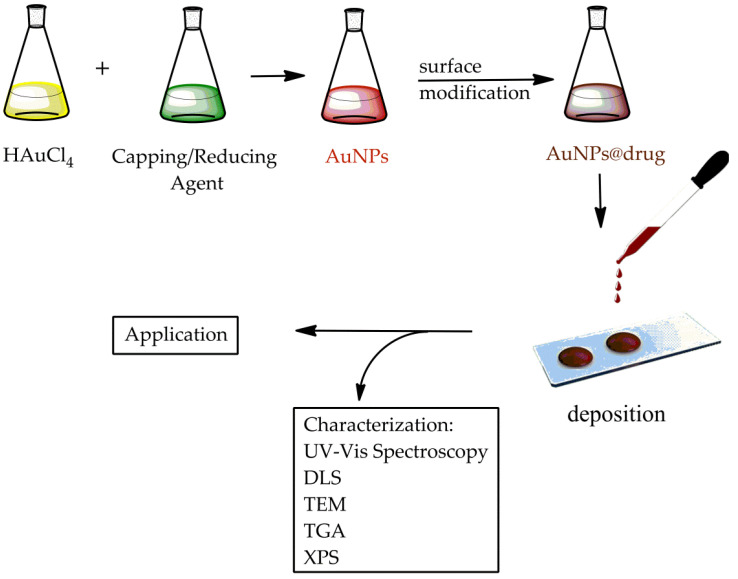
Scheme of the synthesis, characterization, and application of AuNPs.

This review collects the synthesis and chemical–physical characterization methods of AuNPs with interesting shapes that are requested in common applications (Figure 1). Their use in the biology and medicine fields are discussed, both for drug delivery and therapeutic treatments. This work concludes with an overview of all AuNP technological applications that could become a part of everyday life in the near future. 

In particular, AuNPs are employed for medical purposes as:

- Sensors: AuNPs can be used for protein detection in Raman spectroscopy utilized as support for the implementation of the analysis of vibrational energies of chemical bonds [17].
- Probes: used for biological imaging application. AuNPs can produce an array of colors employed in dark-field microscopy [18].
- Diagnostics: AuNPs are able to detect biomarkers as a valid tool in the diagnosis of cancers, infectious agents, and heart diseases [19].
- Treatment Agent Transport: AuNP surfaces can be functionalized with hundreds of biomolecules, which are delivered to target cells [20].
- Photodynamic Therapy: AuNPs generate heat when they are irradiated by 700–800-nm wavelengths of light. The heat of these nanoparticles produced when they are inside cancer cells is then exploited to induce death [21].

Most of the aforementioned products are still restricted to the research and development stages, with human tests, delivery systems, and toxicological assessments that have yet to be analyzed and developed.

## 2. Fabrication of Gold Nanoparticles by Chemical Routes 

The chemical reduction of gold salts into AuNPs consists of two significant phases:

- the choice of proper reduction agents, which provide electrons to reduce gold cations—that is, Au^3+^ and Au^+^ to metallic gold. Nowadays, many reduction agents are used for the synthesis of AuNPs, such as citric acid and citrate, borohydrides, polyols, sulfites, etc. [22].
- the use of stabilization agents, which are crucial to manage the growth of AuNPs in terms of size and geometric shape. In fact, by attributing a repulsive force, they are able to prevent aggregation during the synthetic procedure in the chosen solvent. The most used stabilization agents are sulfur or phosphorous ligands, but polymers and surfactants are also employed; there is also the possibility of using the same molecule to operate as a reducing and stabilizing agent at the same time [23].

The functionalization of AuNP surfaces with specific target molecules is useful to intensify the selective intake in preselected organ cells. AuNP synthesis is highly susceptible to numerous factors, such as solvents, pH conditions, capping ligand exchanges, or the uncontrolled presence of foreign materials. Another relevant aspect is to adapt the sizes and the shapes of AuNPs according to the function they should perform [24]. In the next sections, different physical, chemical, and green synthesis methods of AuNPs, together with their biomedical applications, will be discussed.

### 2.1. Current AuNP Physical and Chemical Synthetic Methods

AuNP synthesis essentially pursues two different approaches:

- a destructive method: top-down approach
- a constructive method: bottom-up approach (Figure 1).

The first approach consists of the separation of bulk materials into nanodimensional particles, adopting different physicochemical methods. Physical methods, like pyrolysis [25], nanolithography [26], thermolysis [27], and radiation-induced methods [28], implicate controlled processes of cutting, milling, and shaping the materials into the desired order and shape. However, the imperfect surface structure of the resulting nanoparticles represents a disadvantage [29]. Another limitation of this approach is the high cost of the process, which requires a massive amount of energy to ensure high-pressure and high-temperature conditions.

In the second constructive approach, AuNPs are synthesized by the self-assembly of single species (atoms, molecules, or clusters) by using chemical or biological methods. This is a cheaper route that provides an enhanced control over the development of the final product with a more homogeneous size, shape, and chemical composition. The bottom-up approach usually consists of wet-chemical synthesis procedures, like chemical [30], electrochemical [31], sonochemical [32], and green synthesis [33]. A limitation of this bottom-up approach is the separation of the synthesized nanoparticles from their reaction mixture, which includes toxic chemicals, organic solvents, and other reagents, except for green synthesis methods. Beyond this common synthesis for the preparation of AuNPs, other current physicochemical approaches are discussed below.

Sputtering is a synthesis protocol consisting of the deposition of a thin layer of nanoparticles that are subsequently subjected to an annealing process. This method is mentioned as the physical vapor deposition (PVD) method [34], and its efficiency principally depends on different factors, such as the substrate type, layer thickness, annealing duration, and temperature. All these variables directly influence the nanoparticles’ sizes and shapes [35].

Micropatterning is a well-known technique similar to a printing process in which a nanomaterial is developed with a specific shape and size through the use of a beam of light or electrons. This is a low-temperature, nonvacuum technique that employs photolithography to synthesize metallic nanoparticles. It is also employed in the development of biosensors, microarrays, tissue engineering, and cellular studies [36]. Lots of lithography methods have been developed, such as colloidal, soft nanoimprinting, nanosphere, and E-beam lithography [37].

Milling is a process that involves the breaking of bulk materials into nanometric structures. In mechanical milling, the reduction of the bulk material in grain size is a consequence of the transferred kinetic energy from the rollers/balls [38]. The shape and the size of the nanoparticles are controlled by various parameters, such as the kind of mill, milling media, intensity, time, and temperature [39]. 

Laser ablation is a faster method that allows the synthesis of nanoparticles with controlled sizes and shapes, resulting in high yields and improved long-term stability [40]. In this process, a pure metal surface is irradiated with a laser beam, causing a low-flux plasma plume, which is then sublimated to produce nanoparticles [41]. The implementation of the laser ablation method in biomedical applications such as the in situ conjugation of biomolecules with gold nanoparticles has been possible thanks to the versatility of the synthesis, which can be carried out in both aqueous and organic solvents. This technique has therefore proven to be more effective than the standard techniques [42].

Pyrolysis is a thermal decomposition technique used individually or in combination with other methods for the synthesis of nanoparticles [34]. This process is the result of an endothermic chemical decomposition that uses heat to break the chemical bonds of the compound, producing metallic nanoparticles and other byproducts in the form of ash [43]. One of the most important issues of this method is the excessive energy consumption.

Chemical vapor deposition is a procedure that consists in the deposition as a thin film of a vapor state reagent on a substrate, together with other gas molecules, thus inducing the overheating of the substrate. During the deposition, the overheated substrate comes into contact with the other gases, bringing about the reduction of the ions [34]. The obtained product is usually highly pure, uniform, and nonporous. This method, however, is disadvantageous, since it is necessary to use special equipment for the production of the films and the reaction chambers; moreover, the gaseous byproducts of this reaction are extremely toxic [2].

Microemulsion is a bottom-up approach method that allows better control over the sizes and shapes of the obtained metal nanoparticles. In general, microemulsion systems are mixtures of two immiscible liquids, one containing the ionic salt and another containing the reducing agent in the presence of a surfactant reagent. The ion reduction is caused by the collision between the two phases [44]. These microemulsion systems are characterized by ultralow interfacial tension, a wide interfacial area, and thermodynamic stability [45]. Nanoparticles produced in the form of emulsions could also be tailored in order to control their sizes and shapes [46]. 

Electrochemical methods for metallic nanoparticle synthesis are usually employed in the biomedical field as biosensors [47]. This technique consists of dissolving a sheet of pure metal in the anode solution to obtain the deposition of the cation on the cathode of an electrochemical cell in the presence of an electrolyte [10]. The effectiveness of this method is influenced by several parameters, such as the type and the concentration of the reducing agent, the type of electrolyte, the purity of the metal, and the stabilizer and the temperature [48]. 

The radiation-induced synthesis method employs ionizing radiation—namely, gamma and X-ray radiations—for the synthesis of highly pure metal nanoparticles [49]. In this approach, an aqueous solution of a reducing and stabilizing agent is exposed to radiation-mediated radiolysis. During this stage, transient molecules are produced, and they are able to reduce metal ions to metal atoms, which aggregate to form nanoparticles. However, the radiation-induced synthesis method presents several critical parameters, including the radiation dose, type of solvent, pH of the system, etc. [50].

Microwave-assisted green synthesis is a fast, easy, and reliable method, which involves the reduction of salts in a surfactant solution and supports the control of nanoparticle morphology [44]. The reduction of ions into metals and, subsequently, into nanoparticles is induced by the heating of the solution. This overheating is due to the fact that microwaves cause an increase in dipole–dipole interactions and, consequently, a better ionic and molecular mobility [51]. 

### 2.2. Turkevich Synthesis 

**Figure 2:**
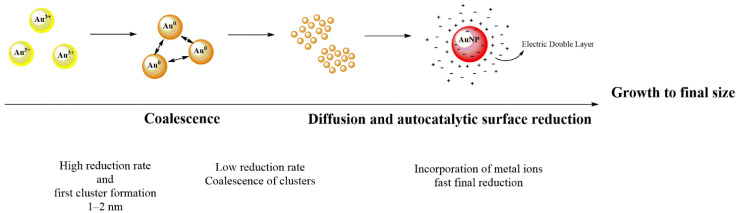
Colloidal gold nanoparticle (AuNP) formation process.

A classical method of AuNP synthesis, introduced by Turkevich in 1951 [52], involves trisodium citrate as a reducing agent. This procedure is carried out considering that some factors, such as the [HAuCl_4_]/[citrate] molar ratio, pH, and temperature, may influence the size and stability of the nanoparticles. The colloidal gold nanoparticle formation process is summarized in Figure 2.

In the first step, metal ions are rapidly reduced, forming metallic clusters of 1 to 2 nm in size. In the second step, the reduction persists, and the newly formed particles undergo coalescence processes by which two or more clusters join each other during contact to create a single, more significant metallic cluster, thus leading to a decrease in the number of particles. Where the average particle size reaches 2.5 nm in diameter, the number of particles remains unchanged, but the particles keep growing in size. Afterwards, the AuNPs begin growing as a result of the incorporation of the gold atoms reduced in the solution. In the third step, when the average radius of nanoparticles reaches nearly 4 to 5 nm, the growth rate drastically increases, and 70–80% of the gold ions, dissolved in the solution, is rapidly reduced [52]. After the synthesis developed by Turkevich, subsequent and several changes have been introduced. For instance, 10-nm AuNPs were synthesized by Yonezawa with a modified Turkevich method by using, as stabilizer agents, sodium 3-mercaptopropionate, and sodium citrate (2.0%) was added (stabilizer/gold ratio is 0.1) [53].

Seitz et al. prepared a single 40-nm gold nanoparticle by mixing HAuCl_4_ and sodium citrate in water under reflux [54]. Yang et al.’s method used HAuCl_4_ in an ethanol solution added into a boiling solution of tri-sodium citrate under vigorous stirring conditions; in this case, the AuNP size was 4 nm [55].

Gold nanoparticles were synthesized by Huang et al. [56], Akiyama et al. [57], and Mayya et al. [58], obtaining an average diameter of the particles of about 10–50 nm.

Ojea-Jiménez et al. [59], investigating AuNP synthesis, demonstrated the influence of the sequence in the addition of the reagents. In the direct method, an aqueous solution of HAuCl_4_ was heated up to 100 °C for 15 min and, finally, added with sodium citrate. During the inverse method, altering the order of the additions of the reagents, the experiment was repeated using the same amounts of reagents but mixing sodium citrate with HAuCl_4_ before the heating step. All the reaction solutions were kept at the boiling point for 5 min before cooling them down to room temperature. The experimental results showed that, while AuNPs synthesized by the direct method had an average diameter of about 18 nm, the indirect method yielded AuNPs with a size of nearly 9 nm.

### 2.3. Synthesis with NaBH_4_ with or without Citrate Addition

In order to perform AuNPs synthesis in milder conditions, in the absence of heating, the Turkevich method was modified with the addition of sodium borohydride (NaBH_4_). Some of these summary procedures are illustrated below. These syntheses were carried out by varying the Au: citrate molar ratios and/or by altering the reaction conditions. The methods are reported in detail in Table 1. 

The AuNP synthesis performed by Zhao et al. involved an aqueous solution of HAuCl_4_ containing NaCl that was cooled and subsequently added to an aqueous solution of sodium citrate and NaBH_4_. In this research work, the AuNPs presented 19 nm in size [60].

Wang et al. prepared nanoparticles with a smaller diameter (around 6 nm, on average) by altering the concentrations of the citrate and reducing agent [61].

### 2.4. Brust–Schiffrin Synthesis 

The Brust–Schiffrin method is one of the most well-known procedures to synthesize spherical AuNPs soluble in organic solvents. The gold surface of nanoparticles presents a high affinity to thiol ligands; this interaction prevents AuNPs from growing and makes possible the formation of small nanoparticles with an average size lower than 10 nm [62].

The “Brust–Schiffrin” method is described as follows: an aqueous solution of HAuCl_4_ was mixed with a toluene solution of tetraoctylammonium bromide (TOAB). The two-phase mixture was vigorously stirred to induce the transfer of tetrachloroaurate into the organic phase. Then, dodecanethiol and sodium borohydride were added to the mixture and vigorously stirred for 3 h. After that, the two phases were separated, and the organic one was evaporated in a rotary evaporator to reduce the volume. Subsequently, with the aim of removing excess thiol, ethanol was added, and the mixture was kept for 4 h at 18 °C. Once the dark brown precipitate was collected by filtering it off and washing it with ethanol, the resulting product was dissolved in toluene and precipitated one more time with ethanol. The AuNPs yielded by this method presented an average size of 2.5 nm [63]. Some syntheses performed by modifying the Brust–Schiffrin method are illustrated below.

Praharaj et al. prepared AuNPs as follows: TOAB was added in toluene and was introduced above an aqueous HAuCl_4_ solution layer. Consequent to shaking the mixture, AuCl_4_^−^ ions were transferred from the aqueous to the organic layer. The gold solution was branched into two vials containing distinct organic compounds: CTAC (cetyltrimethylammonium chloride) and CTAB (cetyltrimethylammonium bromide). Both mixtures were well-stirred, and then, NaBH_4_ was added to each solution. At this point, the reaction mixtures were vigorously shaken. During this stage, a change in the color was observed; firstly, the yellow tone due to the presence of AuCl_4_^−^ vanished, and the solution became colorless; afterwards, it turned into a wine-red color after additional stirring. The final particle size was 10 nm [2]. Kuroda et al.’s AuNP synthesis included the use of MPA (3-mercaptopropionic acid) instead of alkanethiols. An aqueous HAuCl_4_ solution was mixed with a toluene TOAB solution. Then, an aqueous MPA solution was mixed, and then, an aqueous NaBH_4_ solution was promptly added to the mixture, which was stirred overnight. The aqueous phase, containing MPA-protected AuNPs with 2 nm of diameter, was collected. The MPA carboxylic groups were deprotonated into carboxylate groups by sodium hydroxide produced by reducing NaBH_4_, which allows MPA-protected AuNPs to disperse in water [64]. Ghosh et al. prepared AuNPs from a HAuCl_4_ aqueous solution with NaBH_4_ as a reducing agent and DMAP (4-(N,N-dimethylaminopyridine)) as a stabilizer. In a typical synthesis, an aqueous HAuCl_4_ solution was combined with a toluene TOAB solution acting as a phase transfer reagent. This results in the rapid migration of the AuCl_4_^−^ ions to the organic layer within a few seconds. Then, a freshly prepared aqueous solution of NaBH_4_ was added to the mixture and vigorously stirred. Consequent to the addition of the reducing agent, a change in the organic phase color was observed—that is, from light yellow to wine red, within a few minutes. After the separation of two phases, the organic phase was washed with sulfuric acid, sodium hydroxide, and water and consequently dried over anhydrous sodium sulfate. Afterwards, the precipitation of nanoparticles was inducted by adding DMAP to the organic phase, and the excess of the unreacted DMAP was removed by washing the residue three times with toluene. Finally, the precipitate was suspended again in water to collect only DMAP-protected AuNPs with a 20-nm average size [65]. 

Wang et al. proposed their AuNP synthesis, mixing an aqueous HAuCl_4_ solution with a toluene TOAB solution. A color change was observed; the yellow aqueous phase became colorless, while the organic one turned orange. Subsequently, a freshly prepared aqueous NaBH_4_ solution was slowly added into the reaction mixture over a period of 30 min, and the mixture was vigorously stirred for 30 min more. The organic layer was then separated and washed with H_2_SO_4_ and nanopure water. Finally, the organic phase was dehydrated using MgSO_4_ and filtered over a filter paper, yielding 52-nm AuNPs [66]. Kim et al.’s modified synthesis is described as follows: an aqueous HAuCl_4_ solution was added to the toluene TOAB solution and stirred. Chlorobenzenemethanethiol was dissolved in toluene, and NaBH_4_ was added in the water phase. The mixture solution was agitated for 3 h; the toluene phase was separated, and its volume was reduced by evaporation. The AuNPs produced were precipitated into methanol, filtered, and then washed again with methanol. The AuNPs collected had a final size ranging between 3 and 4 nm [67]. Other synthesis procedures are shown in Table 1.

### 2.5. Synthesis by Seeding-Growth Technique

In the proposed AuNP seeded-growth synthesis, a reducing agent is employed to produce in a first reaction step: Au (0) nanoseeds from a gold precursor. In a second reaction step, these nanoproducts are induced to grow into gold nanorods, using the compound as the cationic surfactant, which selectively adsorbs specific crystal facets showing high-surface energies. Usually, the nanorod’s growth is allowed by using small concentrations of additional ions (such as halides) as surface passivation components in a structure-directing role [68]. Jana et al. described, in their paper, a detailed seeding-growth procedure for preparing the AuNPs: an aqueous HAuCl_4_ solution was added to an aqueous trisodium citrate solution. Then, NaBH_4_, the freshly prepared solution, was incorporated under constant stirring conditions. The reaction mixture turned pink rapidly after the addition of NaBH_4_, suggesting the formation of AuNPs. In this case, citrate acted only as a capping agent, since it is not able to reduce gold salt at room temperature. For the growth solution, cetyltrimethylammonium bromide (CTAB) was added to an aqueous HAuCl_4_ solution. After heating the mixture until a clear orange color change was observed, it was cooled at room temperature and employed as a stock growth solution. Then, four 50-mL conical flasks were coded as A, B, C, and D. In set A, the prepared growth solution was mixed with an ascorbic acid solution, and after the addition of the seed solution, the mixture was stirred for 10 min. A change in the color into wine red was observed, and finally, the AuNPs yielded were spherical, with 5.5-nm sizes. Likewise, in set B, both the growth solution and ascorbic acid solution were put together, and the seed solution was added under vigorously stirring conditions for 10 min. The solution’s color changed into a deep red, and the collected AuNPs were spherical, with an 8-nm diameter. In set C, the growth solution was mixed with an ascorbic acid solution, and solution B was added under vigorously stirring conditions for 10 min. The AuNPs prepared following this procedure were approximately spherical, with a diameter of 17 nm, and the final color of the mixture was reddish-brown. The latter was used as a seed in set D, where the growth solution was mixed with a solution of set C and an ascorbic acid solution. The collected AuNPs were both spherical, with 37-nm diameters and rods, with a major axis of 200 nm and a minor axis of 17 nm, and the resulting color of the solution was brown. Overall, the prepared solutions—namely A, B, C, and D—were stable for nearly two months thanks to the presence of CTAB as a stabilizer agent [69]. Sahoo et al. performed a synthesis of AuNPs using the seeding-growth technique. The procedure involved the use of a growth solution of HAuCl_4_, CTAB, acetone, and cyclohexane, which was consequently mixed with AgNO_3_ and ascorbic acid. Then, the growth solution was divided into four parts containing 5 mL each and, subsequently, treated with the seeding-growth technique, as previously described. The mean size of the AuNPs was 10 nm [70]. 

He et al. reported a synthesis using 1,2-bis(10,12-tricosadiynoyl)-*sn*-glycero-3-phosphocholine (PL) for the preparation of the growth solution. The seed solution was prepared with an aqueous HAuCl_4_ and trisodium citrate solution. 

### 2.6. Synthesis by Ascorbic Acid

Ascorbic acid is an eco-friendly molecule well-known for its antioxidant properties in biochemical reactions. It is biodegradable and biocompatible, it has a low toxicity, and it is highly soluble in an aqueous medium. Different papers, reported in the literature, have described its remarkable reduction properties.

The synthesis proposed by Khan et al. can be summarized as follows: HAuCl_4_ was mixed with ascorbic acid and CTAB. Following the color change of the solution, the formation of a precipitate occurred due to the difficulty of complexation and/or solubility of the CTAB. It has been shown that, by reacting solutions of ascorbic acid with solutions of HAuCl_4_ and CTAB at different molar concentrations, AuNPs of irregular shapes with dimensions from 24 to 42 nm were obtained [71]. Polyhedral AuNPs like cubes, tetrahedrons, and octahedrons have increased attention because of their different applications, such as in catalysis, plasmonic, and SERS-based sensors [72]. Boca et al., in their paper, described AuNP chitosan-coated synthesis utilizing ascorbic acid as a reducing agent and the chitosan biopolymer as both a capping and stabilizer agent. The AuNPs obtained had an average diameter of 18 nm [73]. 

### 2.7. Green Synthesis Methods

Recently, AuNP green syntheses have been widely documented in the literature; they consist of alternative eco-friendly and biocompatible procedures performed by using plant extracts. Various chemical compositions and amounts of reducing agents may be found in organic extracts, altering the resulting product. Different geometrical shapes and sizes may be obtained, affecting the function and, thus, the final application. Different amino acids, proteins, enzymes, amines, aldehydes, ketones, carboxylic acids, phenols, flavonoids, and alkaloids can provide electrons to induce the reduction of cationic gold into AuNPs. The properties of the resulting products depend on the concentrations of the plant extract, metal salt, pH value of the reaction mixture, and temperature [14]. Commonly, the procedure for plant extract preparation includes some extra preliminary steps. A few examples are presented below.

Armendariz et al. [74] described the synthesis of AuNPs using *Avena sativa* biomass. Firstly, a sample of oat ground stems was washed with HCl and then rinsed with deionized water; the pH value was adjusted by using diluted HCl and NaOH solutions. In a separate beaker, a KAuCl_4_ solution was prepared and moved to three test tubes containing the oat biomass. While smaller AuNPs were collected at pH values of 3 and 4 (5–20 nm), larger AuNPs were obtained at a lower pH value—namely, 2 (25–85 nm).

Hamelian et al. [75] produced AuNPs using thyme; the plant was washed different times with deionized water and dried at room temperature in the incubator before being ground into powder by means of a mortar. This powder was boiled in water until the color of the solution turned a light yellow. The resulting extract was mixed into an aqueous HAuCl_4_ solution at room temperature and constantly stirred for 1 h to complete the reduction process. The formation of AuNPs was determined by observing the color change of the solution to dark red, which was then centrifuged, and the surfactant was discarded. Subsequently, the collected AuNPs were washed several times with deionized water to remove the excess biological materials, uncoordinated, and, finally, dried at 50 °C in an oven. The AuNPs had diameters that ranged between 6 and 26 nm.

Nazirov et al. [76] suggested a one-pot green synthesis employing the imidazole derivative of chitosan; in particular, the (N-(4-imidazolyl)methylchitosan) IMC solutions were prepared by dissolving the appropriate amount of polymer into an acetic acid solution. The HAuCl_4_ solution was synthesized by the oxidation of a required amount of metallic gold foil in aqua regia and subsequent cycles of evaporation/the addition of concentrated HCl. The HAuCl_4_ solution was combined with IMC solutions, kept at 25 °C, and permanently stirred for at least 7 days. The yielded AuNPs were ultra-small, with an average diameter of 2.3 nm. 

Taib employed a *Hibiscus sabdariffa* L. extract as a reduction agent for AuNP synthesis. The fresh *H. sabdariffa* L. was washed several times with distilled water to clear away its impurities and dried for 2 days at 65 °C. Once the product was dry, it was powdered and dispersed into distilled water for 30 min at 60 °C. The mixture was then separated by filtration, leaving a pale orange color residual extract with a pH of 3.1. This extract was finally used in 9-nm AuNP synthesis [77]. 

AuNPs with an average size of 10–15 nm were synthesized using biopolymer chitosan as a reducing agent. A newly prepared solution of chitosan dissolved into an acetic acid solution was mixed with a HAuCl_4_ solution and stirred at 70 °C until the AuNPs were formed when the color of the solution turned from pale yellow to red [78].

Suvith et al. used *Guggulutiktham Kashayam* (GK), an ayurvedic herbal medicine, as a reduction agent for metallic AuNP synthesis with a size range between 15 and 50 nm. GK was added to an aqueous HAuCl_4_ solution at room temperature, and, in this condition, the synthesis took nearly 1 h for a complete reduction. By contrast, the synthesis of the AuNPs carried out at 100 °C was found to be faster, taking just 1 min [79].

Meena Kumari et al. employed commercial edible coconut oil of high purity as a reducing agent. A solution including coconut oil and acetone was prepared. An aqueous HAuCl_4_ boiling solution was added to different volumes of the aforementioned coconut oil and acetone one. It was then possible to conclude that the smaller the volume of reducing agent solution added, the larger the AuNP size [80].

Sheny et al. used *Anacardium occidentale* (AO) for AuNP synthesis: AO fresh leaf was hydrodistilled using a Clevenger apparatus, producing oil, which was solubilized acetone, to get the reducing agent solution for the synthesis of AuNPs. An aqueous solution of HAuCl_4_ was added to the oil, both at 100 °C and at room temperature. The AuNPs prepared at room temperature were hexagonal in shape, and the average length was 36 nm. The AuNPs prepared in 100 °C were both triangular and hexagonal in shape, with their sizes ranging from 15 to 37 nm [81].

Spherical AuNPs of 15 nm in size were prepared by Philip et al. using natural honey as the reducing agent. An aqueous solution containing 28% (*w*/*w*) of honey and 42% (*w*/*v*) HAuCl_4_ was prepared, mixed, and vigorously stirred [82].

Philip et al. used *Volvariella volvacea* (VV) mushroom as a reducing agent to obtain AuNPs from 20 to 150 nm in size; VV was boiled in water and then filtered with the aim of collecting the VV extract. Different volumes of mushroom extract were added to the same aliquot of HAuCl_4_ solution at two different temperatures—namely, 40 °C and 80 °C [83]. 

Recently, AuNPs with an average particle size of 5–22 nm were obtained by Bonilla-Nepomuceno et al. using an aqueous coffee (*Coffea arabica* L.) pulp extract as a reducing agent. HAuCl_4_ at concentrations of 3, 4, and 5% was added to the extract coffee pulp solution and kept at 85 °C for 20, 40, or 60 min, respectively. In this work, three factors were also considered as the precursor concentration, reducing agent concentration, and reaction time, and their combined effects on the maximum intensity, particle size, and particle density were analyzed [84]. 

Adewale Akintelu et al. obtained AuNPs with antibacterial properties from the *Garcinia kola* pulp extract as a reducing agent. The *Garcinia kola* pulp extract was obtained from the fruit of this tree known in Africa for its medicinal properties [85]. 

Table 1 summarizes the presented scientific papers.

**Table 1:** Summary of the methods for AuNP synthesis.

HAuCl_4_ Concentration	Chemicals Used	AuNPs Size	Reference
Turkevich synthesis			
0.15 mM	sodium citrate	20 nm	[52]
5.8 mM	sodium 3-mercaptopropionate, and sodium citrate	10 nm	[53]
0.5 M	sodium citrate	40 nm	[54]
24.3 mM	sodium citrate	4 nm	[55]
1 mM	sodium citrate	10 nm	[56]
0.25 mM	sodium citrate	18 nm	[57]
1 mM	sodium citrate	13 nm	[58]
0.25 mM	sodium citrate	9 nm	[59]
Synthesis with NaBH_4_ with/without citrate			
0.25 mM	NaCl, NaBH_4_ and sodium citrate	19 nm	[86]
0.1 mM	sodium citrate, NaBH_4_	6 nm	[61]
0.3 mM	NaBH_4_	30 nm	[87]
0.25 mL	sodium citrate, NaBH_4_	3.5 nm	[88]
0.1 mM	NaBH_4_	7 nm	[89]
0.03 mM	sodium citrate, NaBH_4_	4 nm	[90]
0.3 mM	sodium citrate, NaBH_4_	8 nm	[91]
Brust–Schiffrin method			
30 mM	tetraoctylammonium bromide, dodecanethiol, NaBH_4_	2.5 nm	[63]
30 mM	tetraoctylammonium bromide, NaBH_4_	3.4 nm	[92]
10 mM	tetraoctylammonium bromide, cetyltrimethylammonium chloride, cetyltrimethylammonium bromide, NaBH_4_	10 nm	[2]
30 mM	tetraoctylammonium bromide, pentanethiol, NaBH_4_	5 nm	[93]
10 mM	3-mercaptopropionic acid, tetraoctylammonium bromide, NaBH_4_	2 nm	[64]
30 mM	4-(N,N-dimethylaminopyridine), NaBH_4_	20 nm	[65]
0.45 mM	tetraoctylammonium bromide, dodecanethiol, NaBH_4_	1.8 nm	[94]
34 mM	tetraoctylammonium bromide, NaBH_4_	1.8 nm	[95]
30 mM	tetraoctylammonium bromide, NaBH_4_	10 nm	[96]
0.1 mM	tetraoctylammonium bromide, 1-decanethiol, NaBH_4_	4 nm	[97]
30 mM	tetraoctylammonium bromide, 4-dimethylaminopyridine, NaBH_4_	5.5 nm	[98]
4.0 mM	tetraoctylammonium bromide, Chlorobenzenemethanethiol, NaBH_4_	3–4 nm	[67]
50 mM	HCl, NaBH_4_, NaOH, dodecanethiol, n-hexane,	4 nm	[99]
5 mM	tetraoctylammonium bromide, 1-hexanethiol, NaBH_4_	2 nm	[100]
Synthesis by Seeding-Growth technique			
0.25 mM	sodium citrate, cetyltrimethylammonium bromide, NaBH_4_, ascorbic acid	6 nm 17 nm 37 nm	[69]
0.25 mM	cetyltrimethylammonium bromide, AgNO_3_, ascorbic acid	10 nm	[70]
10 mM	cetyltrimethylammonium bromide, NaBH_4_, AgNO_3_, ascorbic acid	33 nm length 13 nm width	[101]
0.25 mM	1,2-Bis(10,12-tricosadiynoyl)-*sn*-glycero-3-phosphocholine, sodium citrate, NaBH_4_, ascorbic acid	17 nm	[102]
Synthesis by ascorbic acid			
0.05 mM	cetyltrimethylammonium bromide, ascorbic acid	30 nm	[71]
20 mM	cetyltrimethylammonium bromide, ascorbic acid	15 nm	[72]
0.5 mM	ascorbic acid, chitosan	18 nm	[73]
Green synthesis			
0.1 mM	*Avena sativa biomass*	5–20 nm	[74]
1 mM	Thyme	6–26 nm	[75]
10 mM	N-(4-imidazolyl)methylchitosan	3 nm	[76]
5 mM	*Hibiscus sabdariffa* L.	9 nm	[77]
1 mM	Chitosan	10–15 nm	[78]
0.2 mM	*Guggulutiktham Kashayam*	15–50 nm	[79]
0.3 mM	coconut oil	25–45 nm	[80]
0.25 mM	*Anacardium occidentale*	15–40 nm	[81]
0.1 M	Natural honey	15 nm	[82]
60 mM	*Volvariella volvacea* mushroom	20–150 nm	[83]
3–5%	Aqueous coffee pulp extract (*Coffea arabica* L.)	5–22 nm	[84]
1 mM	*Garcinia kola* pulp extract	18–38 nm	[85]

## 3. Gold Nanoparticles Surface Modification

**Figure 3:**
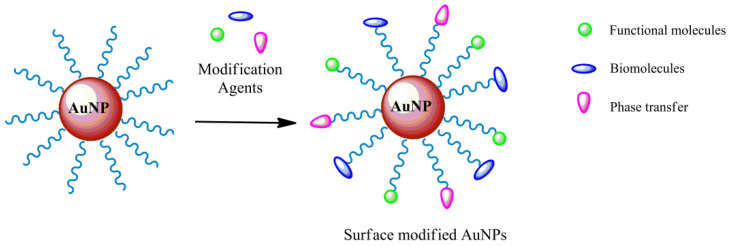
Multi–functional modification of AuNPs surface.

Different methods for the surface modification of nanoparticles have been described, such as secondary modification, chemical reduction, green synthesis, microbial, and microwave-assisted methods [103]. The chemical reduction method is the most frequently used for the surface modification and functionalization of AuNPs with a specific ligand (Figure 3), such as biomolecules, functional molecules, and phase transfer. This procedure implements the analytical, chemical, and biological properties of AuNPs, making them useful for multiple applications [104,105]. 

In several applications, AuNPs behave as nanocarriers, and, therefore, they are chemically modified for their specific functions. The suitable surface functionalization of AuNPs for every application is of great relevance. The specific functionalization can enhance the stability and biocompatibility, as well as preventing their aggregation. The surface modification of AuNPs mainly serves several functions: (i) ligands, attached on the AuNP surface, support their stability, (ii) linkers, bonded on AuNP surfaces, allow additional functionalization reactions, and (iii) functional ligands and biomolecules, directly immobilized on AuNP surfaces, allow further functionalization or bioconjugation, providing a more comprehensive application range [106]. The excellent colloidal stability of the AuNP solution is achieved when mechanisms of electrostatic and steric repulsions dominate. The colloidal stability is an important property that prevents the aggregation of AuNPs that causes the loss of their functionalities. In fact, AuNP stability is mainly based on electrostatic repulsion when ligands or capping agents are charged. The strength of electrostatic repulsion is connected to the degree of the surface charge, which, in turn, depends on the pKa value of the ligands, the pH value, and the ionic strength of the solution. Thus, capping agents and ligands, with high surface charges such as citrate and ionic polymers, are generally used as stabilizing agents [107]. Surface modification using long-chain spacers, such as PEGylation and neutral polymers, is a method to shield and support the stability of AuNP colloidal suspensions, depending on steric repulsion [108]. The ligand amount and direction are two further critical factors of the AuNP properties, since they play a key role in the strength of the interactions with other molecules. Additionally, the environmental conditions are important; for instance, purified AuNPs stored at room temperature may aggregate within 6 days, whereas AuNP storage at 4 °C in the dark extended the AuNP stability up to 20 days [109]. Thus, the morphology of the coating of functionalized AuNPs might change because of the dynamic nature of the AuNPs in relation to the storage time and temperature [110].

### 3.1. Secondary Modification

**Figure 4:**
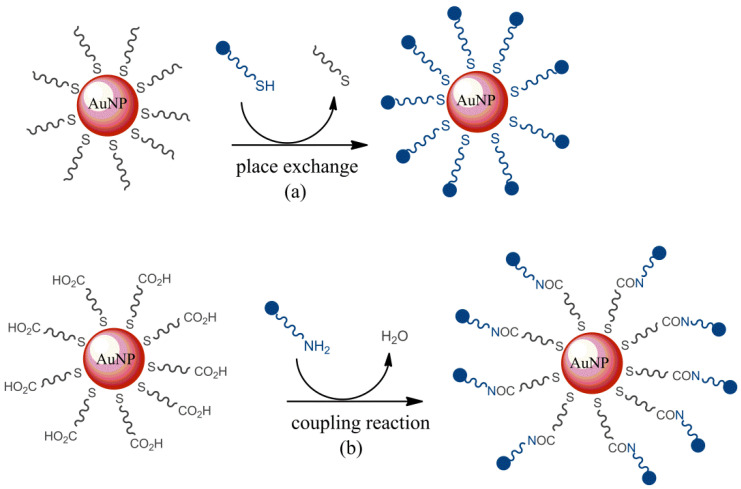
Representation scheme of the surface functionalization method through (**a**) a place exchange and (**b**) coupling reaction.

The secondary modification technique is based on the “place exchange” of a thiol ligand to incorporate various surface functionalities on AuNPs. A literature survey showed several works proposing the functionalization of AuNP surfaces. The thiol exchange ligands chosen can vary to a great extent, including organic/inorganic dyes, smart polymers [111], biomolecules [112], and drug molecules [113]. Different ligands, such as ferrocenyl hexanethiol, ferrocenyl octanethiol, ferrocenyl methyl acrylate, and ferrocene thiophenol, were also employed for electrochemical applications [114]. These nanocomposites are able to host different anions and to enhance the redox properties or the sensitivity in anion detection [115]. The secondary modification methods are represented in Figure 4. The secondary reaction step on the AuNP surface occurs through a chemical coupling reaction [116], polymer formation [117], and electrostatic and selective interactions [118]. Two well-known methods for the modification of AuNP surfaces are coupling and esterification. Indeed, carboxylic acid terminated the thiol ligands grafted on AuNP surfaces, giving rise to amides or esters by a condensation coupling reaction with molecules containing an amine or hydroxyl group. This secondary modification approach allows generating different functionalities by a condensation reaction. 

### 3.2. Physical Sorption

AuNP incubation with specific ligands is a simple method for the rapid immobilization of functional molecules. Functionalization by the physical sorption of ligands or biomolecules on AuNP surfaces is essentially driven by electrostatic and hydrophobic interactions. Thus, the control of the pH value of the incubation solution is essential, since it modifies the AuNP charge state and the degree of immobilization of the functional molecules [119]. In fact, if biomolecules denature during immobilization, they will permanently lose their bio-functionality. A specific buffer solution is then employed to maximize the attractive electrostatic interactions between AuNPs and the ligand in an electrostatically driven adsorptive immobilization method. Despite being a simple procedure, adsorptive immobilization is not always the best approach [120]. Presnova et al. synthesized streptavidin@AuNPs using both adsorptive immobilization of the protein driven by electrostatic interactions (pH 7) and covalent-binding procedures [121]. These biomolecules were employed as markers for the detection of single hybridization events of biotinylated DNA and oligonucleotides on a silicon surface with a single oligonucleotide strand. The study results reported that only streptavidin–AuNPs conjugates, synthesized by covalent binding, could be employed for the adequate detection of oligonucleotide hybridization, whereas streptavidin@AuNPs conjugates, prepared by electrostatic interaction, separated into their original components during the washing stage. The conjugation effectiveness of the direct connection is then dependent on the incubation conditions, such as the concentration and molar ratio of AuNPs and the ligand pH values. In addition, adsorptive immobilization brings about a random orientation of the immobilized biomolecule or ligand, so only a part of them may be active as long as some active sites may be partly or fully [122].

### 3.3. Dative Bond and Formation of “Self-Assembled Monolayers” (SAMs)

**Figure 5:**
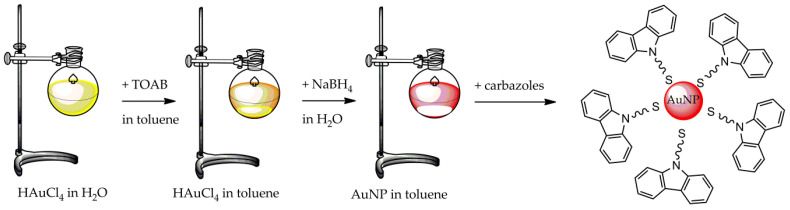
General procedure of synthesis of AuNP@N-thio-alkylcarbazoles [125].

Surface modification based on a dative bond among metallic atoms of the AuNP surface and the ligands, including available sulfur, oxygen, or nitrogen atoms, by giving their unshared electron pairs is one of the most-used procedures for ligand or biomolecule functionalization on AuNPs [123]. Sulfur, as a thiol group, is the preferred atom because of the coordination bonds between gold and the thiol group, which have a bond energy of nearly 40–50 kcal/mol and, thus, are comparable to gold–gold bonds [124]. In fact, thiolated ligands bond on the AuNPs surface in a dense organization, producing self-assembled monolayers, which are simple and stable, allowing an opportunely oriented immobilization of ligands. In a typical surface modification procedure, bifunctional linkers containing a terminal thiol group on one end and a second terminal functional group on the other are often employed [125]. The thiol group links AuNP surfaces, while the reactive functional group protects functionalized AuNPs, preventing the covalent coupling of ligands or other biomolecules [105]. An example is shown in Figure 5, with hydrocarbon chain-functionalized carbazole molecules containing a thiol group on the tail behaving as capping agents for AuNPs [126]. 

Bifunctional linkers with spacers like PEG are commonly used for AuNP surface modification because of the ability of PEG to sustain colloidal stability and decrease the nonspecific binding of proteins from the environment. Furthermore, its terminal reactive functional group can be used for the coupling of peptides or proteins [127]. The direct connection of biomolecules without a spacer or linker with site-specific bioconjugation is possible through a sulfhydryl group from a cysteine. *Cys*-linked proteins have been immobilized following the procedure described by Jeong et al. [128]. Contrarywise, active sites may be partly blocked if biomolecules are randomly oriented on AuNPs because of the hindered accessibility of the active sites, leading to a decrease in the binding capacity or a reduction of the enzyme activity. This straightforward modification via SAMs can be exploited as a sample preparation strategy, such as capturing and enhancing analytes containing a thiol group. Faccenda et al. employed unmodified AuNPs to isolate peptides containing SH groups, exploiting the Au–S bond formation from the sample solution [129,130,131].

### 3.4. Polymer Coating

The employment of polymer coatings as spacers for ligand immobilization is a popular approach for AuNP surface modifications. The aggregation of AuNPs driven by Van der Waals interactions is prevented thanks to the steric hindrance caused by the long-chain structure of neutral polymers yielding more stable AuNPs [132]. Ionic polymer coatings are also used because of their ability to enhance repulsive electrostatic interactions and improve the colloidal stability of AuNP surfaces due to their highly abundant charges. Moreover, a great number of reacting functional groups contained in neutral polymers and polyelectrolytes after their immobilization provides lots of reactive sites on AuNP surfaces for further functionalization [133]. The immobilization of nanoparticles through sol–gel synthesis can be found in a series of technological applications, such as coatings, catalysis, filters, and optical materials, and so on. The sol–gel synthesis of AuNP core–shell systems with different protective functionalities preserves their colloidal stability and allows full control of the morphology and properties of the nanoparticles [134,135,136,137,138,139].

### 3.5. Covalent Immobilization of Ligands

The typical covalent immobilization of ligands on AuNPs is carried out through a two-step approach. In the first step, the AuNP surface is preactivated with reactive functional molecules, such as carboxylic acid or amine groups, often introduced via SAM immobilization of bifunctional linkers containing a reactive thiol for inducing the formation of an Au–S bond on one end and the other reactive functional group on the other end [140]. Similarly, a polymer or polyelectrolyte coating can be used for the formation of the AuNP surface, layer-by-layer, improving the presence of the reactive functional groups able to immobilize targeted molecules covalently [141]. The covalent attachment of ligands usually achieves well-known bioconjugation chemistries from peptide and protein chemistry by amide coupling, such as the D- and L-configuration of poly(N-acryloyl-valine) polymers covalently grafted on AuNPs, to study the polymer chirality effects on the interactions among proteins and AuNP [142]. A layer-by-layer polyelectrolyte coating method was used for AuNP surface modification with cationic polyallylamine and anionic polystyrenesulfonate. A large number of surface-exposed amino groups, available after the polyallylamine layer coating, were used to graft an antibody through its carboxylic acid groups, covalently [132]. Then, as well, Liu et al. synthesized AuNPs based on nanobioreactors for antibody digestion to 50-kDa fragments using papain as the enzyme [141]. The latter was bonded via amide coupling to AuNPs functionalized with a polyacrylic acid layer by their carboxylic functional groups. An additional covalent immobilization procedure was described by Haller et al. [143] that used glycan residues of antibodies for connecting them to AuNPs. In the first step, the glycan residues were oxidized with IO_4_^−^ to aldehyde groups. In the second step, the aldehyde groups were derived with a bifunctional thiolated PEG linker containing a terminal hydrazide group. Then, the protein conjugate was trapped on AuNPs through the Au–S bond, producing a highly stable AuNPs@antibody conjugate, which was oriented and had a higher binding capacity of antigen compared to that of the antibody, leading to a random orientation. 

Fullerene has been commonly used for the synthesis of composite materials due to its mechanical, spectral, and structural properties. In many research works, different thiolated fullerenes with alkyl chains have been employed to stabilize AuNPs and tested for electrochemical or photoelectric applications [144]. Carbon nanotubes and AuNP composites are highly favorable for diverse applications, such as optics, electronics, biosensors, and catalysis [145]. Various methods have been tested to functionalize carbon nanotubes by AuNPs, such as thermal decomposition, and the pre-functionalization of the nanotube surfaces with carboxylic acid groups, amino groups, or other functional groups that easily connect AuNPs, implementing their properties [146]. 

### 3.6. Bioaffinity Immobilization of Ligands

Biomolecule and ligand immobilization through affinity binding is a further method for the synthesis of chemically stable protein@AuNP conjugates. Common high-affinity binding couples consisting of protein A/G–immunoglobulin, streptavidin–biotin, and antigen–antibody molecular recognition systems have been employed for this purpose. In a series of applications, the bio-affinity of biotin and streptavidin has been widely used for the immobilization of ligands to the AuNPs surface. The low dissociation constant between biotin and streptavidin, which is in the order of *K*_d_ = 10^−15^ mol/L, facilitates the essentially irreversible immobilization of biotinylated ligands on streptavidin–AuNP conjugates. This method allows biotinylated nucleic acids [147], antibodies, and aptamers to be immobilized. A further valid method for the immobilization of antibodies by protein A or protein G, which are antibody-binding proteins, shows a great affinity for a fragment of the various classes of antibodies [133]. A specific binding orientation commonly produces implemented binding capacities of antigens related to AuNPs@conjugates with the random orientation of the immobilized antibody, as often produced by adsorptive immobilization and covalent linkage through AuNPs@carboxy-pegylated. Protein A or Protein G@AuNP conjugates are typical platforms for the immunoaffinity capture of monoclonal antibodies and, also, for the immobilization of antibodies for biomarker extraction [140].

## 4. Chemical–Physical Characterization of Gold Nanoparticles

AuNPs can be characterized by a great number of optical and physical methods. The specific optical and physical characteristics of AuNPs are basically caused by the different sizes, morphology, shapes, and colloidal stability of the AuNPs. Therefore, exhaustive characterization with various techniques is of great importance for the quality control of the produced AuNPs. To prevent ambiguous interpretations, a sequence of multiple techniques is generally suggested for suitable and complementary characterizations [148].

### 4.1. Ultraviolet–Visible Spectroscopy (UV-VIS)

Ultraviolet–visible spectroscopy is one of the most common techniques for the characterization of the localized surface plasmon resonance (LSPR) of AuNPs [149]. LSPR involves the resonance phenomenon of conduction electron oscillation on a metal surface as the response of incident light interactions. In this specific situation, it is limited to the nanoparticle that has a similar or smaller size than the wavelength of light employed to excite the plasmons. A characteristic of LSPs is the optical absorption maximum at the plasmon resonant frequency for AuNPs corresponding to a wavelength of around 520 nm. Thus, the LSPR band is susceptible to changes in size, distribution, aggregation, and surface modifications. Then, this is an easy method for the analysis of the size and distribution of AuNPs, which do not require sample pretreatment. There is a linear dependency of the logarithm of the absorption coefficient (ε) on the logarithm of the AuNP diameter *D* (ln ε = 3.32·ln *D* + 10.81) [97]. In a recent paper, UV-Vis spectroscopy was used as a quantitative method on AuNPs functionalized or not. AuNP concentrations can be estimated from the derived absorption coefficient in accordance with the Lambert–Beer Law from the measured UV absorbance at a wavelength corresponding to the SPR optical absorption maximum (around 520 nm) [150]. Once the particle size is obtained, the corresponding extinction coefficients can be determined, and the related concentration of AuNPs can be calculated using the Lambert–Beer Law [97]. Since bigger AuNPs have a shifted absorbance maximum, it is also possible to correlate the width of the LSPR band with the polydispersity of the AuNP solution [151]. Furthermore, UV-VIS spectroscopy measurements can be used in the evaluation of the aggregation of AuNPs. In fact, when AuNPs aggregate, a shoulder is observed at a wavelength of about 600 nm, close to the characteristic SPR band. All-in-all, this spectroscopic method is a simple and fast analysis technique for the quality control directly after their synthesis without sample pretreatment. Thus, the combination of UV-VIS spectroscopy with other analytic methods is fundamental for an accurate particle size characterization [152].

### 4.2. Dynamic Light Scattering (DLS)

Dynamic light scattering (DLS) is an appropriate and nondestructive technique to obtain the hydrodynamic size distribution of AuNPs and to determine their aggregation state [153]. Having the feature be conducted in situ without a particular sample pretreatment, a DLS analysis was used as an effective tool for biomolecular-binding investigations [154] and, also, the diagnosis of the AuNP conjugate aggregation state by checking the average size variation of the AuNPs as a consequence of ligand binding, and aggregation events have been investigated through this technique. As DLS analyzes Rayleigh scattering from the Brownian motion of the AuNPs, the determination of the AuNP size distribution depends on the intensity of the fluctuations of the scattered light at a certain scattering θ. Unfortunately, the results of the AuNP size analysis using the DLS technique may be easily partial, and the size of the AuNPs measured is slightly overestimated compared to that determined by the TEM analysis. This is because DLS measures the hydrodynamic diameter, and since, the nanoparticle suspended in water is surrounded by an aqueous double layer, its apparent dimensions are altered. The scattering produced by such small particles is isotropic and the intensity of the scattered light (I) is proportional to D^6^, where D is the diameter of the particles (I ~ D^6^). This relationship is valid for small systems whose diameters are less than 1/10th of the laser wavelength (λ). AuNP diameter distributions appear to be different when the measurements are based on the intensity, volume, and number distributions. Moreover, DLS measures the hydrodynamic diameter instead of the dry state diameter like TEM. The DLS technique is useful when it is necessary to analyze the state of particles in a solution, and what is more is that the shell thickness around AuNPs could be determined by comparing the hydrodynamic diameters of AuNPs before and after functionalization [153].

### 4.3. Transmission Electron Microscopy (TEM)

Transmission electron microscopy (TEM) is a fundamental technique used in nanoscience, because it furnishes high-resolution images of AuNPs at the nanometer level. TEM images consent to determine the sizes and shapes of AuNPs in the dry state. Reliable size measurements and the clear structural morphology of AuNPs can be observed [153]. Nevertheless, some substantial information of the AuNPs, such as colloidal stability, are inaccessible by a TEM analysis. In addition, this technique requires sample preparation that may cause some artefacts, such as AuNP aggregation [152]. Moreover, modifications of the surface modifications are not detectable by the standard TEM technique; this would require special equipment, such as cryo-TEM [155], glycerol spraying/low-angle rotary metal shadowing TEM [133], and others.

### 4.4. Thermogravimetric Analysis (TGA)

The physicochemical characterization of nanomaterials needs a higher number of analytical techniques to understand the morphology and composition of the nanoparticles. In fact, their surface compositions may change due to exposure to biological fluids or other environmental factors. When the nanoparticles are modified on the surface, the total and uniform coverage of the nanoparticle needs to be guaranteed; it is therefore necessary to accurately characterize the surfaces and functional coatings on the nanoparticles [156]. TGA is a simple analytical technique that can be reliably used to assess the purity of nanomaterials. During the thermogravimetric analysis, the mass of the sample is monitored as a function of the heating suffered, and the result is a decomposition curve whose analysis gives the oxidation temperature and the residual mass of the sample. For nanomaterials, the residual mass could be due to inorganic residues, metal catalysts residual from synthesis, or impurities inside the sample. The most significant drawbacks of TGA are that it is a destructive technique, and it can be excessively expensive. This characterization method can be used to analyze the amount of organic residues, surface melting properties, and the resistance to oxidation [157]. 

### 4.5. X-ray Photoelectron Spectroscopy XPS

X-ray photoelectron spectroscopy (XPS) is a highly specific analytical technique for the characterization of the chemical composition of surfaces of a wide variety of materials. It is a fundamental tool for analyzing the surface atomic layers and surface chemical structures of AuNPs within several nanometers of the surfaces [158]. Some of the XPS applications include a surface functionality analysis of the organic and plastic coatings and determination of the oxidation state of the catalysts and nanomaterials. Moreover, this technique provides good quantitative and qualitative information, and it is not a destructive technique, and it is compatible with many types of samples: gaseous, liquid, or solid [159]. In relation to the study of the coordination of ligands on the surfaces of nanoparticles, XPS provides interesting (useful) information on the coordination chemistry of the relative ligand with respect to the uncoordinated one.

## 5. Surface Coating Determination

The characterization of the AuNP surface coating is an essential tool for the quality control and regulatory approval of the AuNPs for specific applications in the analysis and biomedical sectors. It is also important for determining the catalytic efficiencies of bionanocatalysts. Quantitative methods developed for the characterization of AuNP shell thickness, ligand orientation, and the number of active parts on AuNPs are briefly summarized in the following sections [160].

### 5.1. Indirect Methods

Indirect methods are extensively employed for determining the ligand densities on AuNP surfaces by quantification of the free ligand in the reaction mixture, such as the supernatant after the functionalization reaction of the AuNP surface [161]. Then, the surface coverage density can be calculated as the difference between the total concentration of the ligand added to the gold solution and the concentration of free ligand after immobilization on the AuNP surface [133]. Depending on the structure and properties of the ligands, different techniques have been developed to determine the amount of unreacted free ligands in the reaction mixture with different detection sensitivities [162]. For instance, hydrazide-functionalized ultrasmall AuNPs (a 1.2-nm core diameter) were produced with excellent stability for catching *N*-glycosylated peptides. The surface hydrazide densities were quantified with a UV detection technique. For this reason, the hydrazide groups linked to AuNPs were protected with an excess of 4-nitrobenzaldehyde (4-NBA); the solution was then centrifuged, and the NBA-coupled AuNPs were removed from the solution. The supernatant was analyzed by UV spectroscopy at 265 nm to quantify the concentration of the unreacted amount of free NBA. The immobilized hydrazide groups were determined by back calculation. Xia et al. described four different methods for the quantification of the surface coverage of the spacer (HS-PEG-NH_2_) on AuNP conjugates: two indirect methods named (i) the fluorescamine-based assay and (ii) ninhydrin-based assay and two direct methods: (iii) the FITC-labeling assay and (iv) Cu^2+^-labeling assay [163]. Fluorescamine and ninhydrin were used as the fluorescent and chromogenic agents in methods (i) and (ii); they are used for the derivatization of the unbound HS-PEG-NH_2_ molecule in the supernatant. These two indirect methods may be imprecise if the reaction is carried out with a large excess of ligands [163].

### 5.2. Direct Methods

Direct quantification methods furnish more precise and accurate results compared to indirect methods. Usually, both the AuNPs and the ligand density concentration are in relatively low ranges. Thus, for accurate quantification, highly sensitive detection assays are required. To develop a sensitive technique, a different mark is used before the quantification analysis. Xia et al. directly detected an “active” –NH_2_ surface shell using FITC or DOTA containing Cu^2+^ to label the terminal –NH_2_ groups of the surface ligand. The marked ligands were detected by the ICP-MS technique or fluorescence after dissolving the AuNPs conjugated in aqua regia. In another example, the thiol ligand density of AuNPs conjugated with different bifunctional thiolated ligands was quantified by ICP-MS measurement through the simultaneous detection and quantification of AuNPs and the thiol group [164]. The experiments were carried out with a set of AuNPs of various diameters for a series of distinct bifunctional ω-thiolated carboxylic acids with PEG and alkyl spacers of different lengths. A linear correlation between the Au:S ratio and the AuNP sizes was observed, with the slopes of these lines correlated to the surface ligand coverage of each particle set. Since this technique is independent from the AuNP concentration, a possible loss of the compound during sample preparation does not constitute a problem. Furthermore, the surface coverage density depends on the spacer length.

## 6. Physical and Chemical Properties of AuNPs Depending on Particle Size

When the AuNP size is gradually reduced, below 10 nm, important differences in the physical and chemical properties can be observed. The most critical result of the reduction in the particle size is the development of the surface/volume ratio. The modifications involve: (i) a lowering in the melting temperature and (ii) a decrease of the interatomic distance [165]. Surface atoms experience an inwards force that is not compensated for in the opposite direction, leading to a decrease of the interatomic distances. This condition allows atoms to have a larger attitude to vibrate around in their positions, thus causing a lowering of the melting temperature. Theoretical studies demonstrate that changes in the electronic structure influence the optical responses of AuNPs, and the lowering of the size at the nanometer level results in different colors, shown by the colloidal dispersion of AuNPs. The metallic character decreasing with the reduction of the AuNP size is due to the shrinkage of the electron energy levels and the formation of a gap between the valence and conduction bands [166]. 

### 6.1. Antibacterial Activity

**Figure 6:**
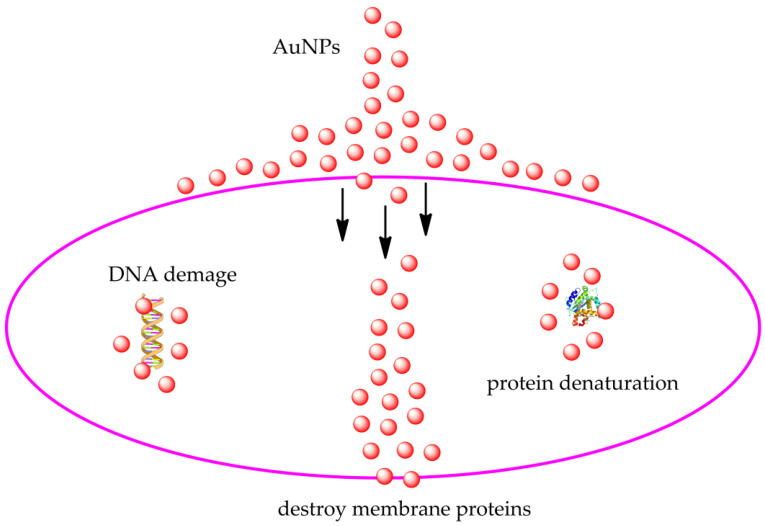
Various mechanism of antimicrobial activity of AuNP@antibiotics/antibacterial coatings.

Due to the increase in the clinical resistance to bacteria, antibiotics have started to lose their bacteriostatic efficacy. Indeed, the hydrophilic nature of antibiotics causes the passage of proteins through channels and pores of the bacteria membrane, resulting in a shorter stay inside the cells and a weakening of their bactericidal effect [167]. The combination of AuNP with antibiotics inhibits its penetration into cell walls, significantly improving its antibacterial activity. Lu’s group synthesized and tested AuNPs @ vancomycin, demonstrating a better antibacterial activity compared to that of free vancomycin. Vancomycin allows a better absorption of bacteria on AuNP @ vancomycin particles. In particular, the bacterial strains on which it shows a better activity (99% in the first 5 h) are Gram-positive (*S.* *aureus*) and Gram-negative (*E. coli*) bacteria, with a minimum inhibitory concentration starting from 30 nmol mL^−1^ [168]. Khandelwal and his group synthesized AuNP functionalized with cefradin [169] and gentamicin sulfate [170]. The experiments carried out by the research group showed that the inhibition by AuNP is a consequence of the direct contact that induces the breakdown of the cell wall and not from the generation of reactive oxygen species (ROS). Wang et al. dealt with the synthesis of Fe_3_O_4_ magnetic nanoparticles loaded with AuNP and streptomycin. The latter exhibited good antibacterial activity against Gram-negative *E. coli* and Gram-positive *S. aureus* bacteria, indicating a good synergy between Fe_3_O_4_–AuNP and antibiotics [171]. The main antibacterial mechanisms of AuNPs are shown in Figure 6. The different methods of synthesis of AuNPs lead to the formation of nanoparticles having disparate sizes with different distributions. The size and surface functionalizations of AuNPs affect their antibacterial activity. By changing the morphology, structure, and dispersion of gold nanoparticles, their antibacterial efficacy can be increased [133]. Gold nanoparticles have been extensively studied and used as an effective antibacterial agent thanks to their stability, low toxicity, high specific surface area, and easy functionalization [172]. In general, AuNPs with a small diameter in the range of 2–15 nm are mostly used in immunology, biochemistry [173], and high-power microscopy [174], while AuNPs with an average diameter between 20 and 60 nm are used for environmental tests, DNA tests, and in the administration of drugs [175]. As regards the largest AuNPs, whose diameters are in the range of 80–250 nm, they are mainly employed in medical, electrical, and X-ray optics [176]. Vanaraj’s group studied the antibacterial activity of AuNPs with a diameter of 100 nm synthesized by means of a methanolic extract of *C. ternatea* leaves. In particular, the inhibition of the rate of formation of *Pseudomonas aeruginosa* by 94.4% was observed when the AuNP concentration reached 100 μg/mL [177]. Lanh and his colleagues tested 10-nm diameter gold nanotubes against *E. coli*, *S. Typhimuriumm*, *S. aureus*, and *L.* *monocytogenese*, showing how the antibacterial activity is favored at the minimal concentrations of 0.05 μg/mL, 0.2 μg/mL, 0.008 μg/mL, and 0.0002 μg/mL, respectively [178]. 

### 6.2. Localized Surface Plasmon Resonance in Nanoparticles (LSPR)

**Figure 7:**
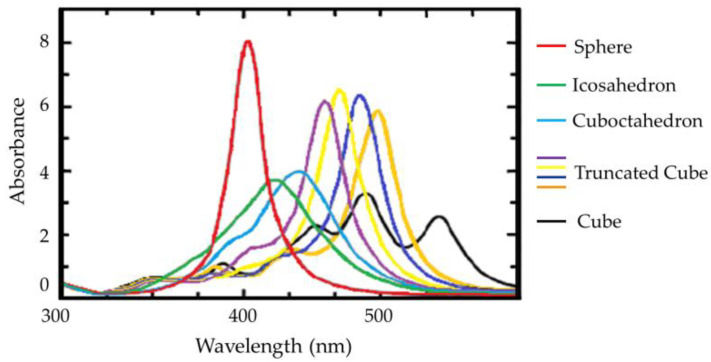
UV–Vis spectra for gold nanoparticles of different shapes.

Surface plasmon resonance (SPR) supports different standard devices for measuring material adsorption onto metal nanoparticles. SPR is defined as the resonant oscillation of conduction electrons excited by incident light. In AuNPs, the electron oscillation is dissimilar from the SPR. If AuNP sizes are around 20 nm—that is, smaller than the wavelength of the electromagnetic wave—the AuNP electron cloud is detected by the electric field. Thus, the entire group of electrons is polarized, inducing the formation of a dipole moment in the nanoparticle’s surface, and, as a consequence, this oscillating polarization generates an electric field opposite to that of excitation, resulting in a restoring force. This oscillation damping takes place following two different pathways: heat production and light scattering. LSPR is a bulk effect occurring in a nanometric volume of metals. The principal characteristics of LSPR can be summarized as follows: (i) the plasmon resonance is displayed in the visible or near-infrared spectral ranges for AuNPs that display light scattering, with a cross-section wider than conventional dye [179]. (ii) The AuNP standard shift for a molecular interaction is about 10 nm of magnitude, and the LSPR is strongly related to the surroundings of nanoparticle surface. The plasmon amplification is extensively employed for improving the sensitivity of biosensors. The plasmon redshift is commonly caused by the aggregation of AuNPs in such colloidal solutions. This plasmon intensification is circumscribed close to the particle, and it is exploited as an optical nano-antenna. The AuNP size does not affect the LSPR band position, and the AuNP volume exclusively influences its intensity. In biosensor application, AuNPs are excited by using a light source compatible with the biological window between 650 and 900 nm. The LSPR can be modified: (i) by changing the reagents and the solvent surrounding the AuNPs; (ii) by modifying the AuNPs shape, such as cube, ellipsoids, icosahedra, triangle, etc.; and (iii) by employing core–shell nanoparticles. All these factors may shift the LSPR to higher wavelengths. For nanoparticles with different shapes, the absorption and scattering field can be related to the polarizability and, thus, to the shape, such as ellipsoids [180] and cylinders. For example, in the case of ellipsoid nanoparticles, plasmon resonances correspond to the oscillation of surface electrons ahead of the three axes of the ellipsoid. LSPR can be shifted up to the infrared range by changing the lengths of the axes and the direction of the light beam and its polarization. When such objects are obtained with inhomogeneous sizes and shapes, the optical response is an average resulting from the individual optical responses of AuNPs of various sizes and orientations in space. For a spherical nanoparticle, all resonances have degenerated, and the depolarization factors are equivalent. The plasmon modes of AuNPs with complicated shapes can be determined by using computational methods, such as calculations that have been done to assimilate nanorods in ellipsoids, showing a more complicated plasmonic structure [181]. The number of plasmon modes raises the more the shape diverges from a sphere and according to the number of vertices constituting the nanoparticle. Different plasmon modes are plotted in Figure 7. Plasmon resonance is blue-shifted when the nanoparticle shape becomes more symmetric. If the particle is bigger than 60 nm, larger bands are observed and tend to shift the dipolar contribution [182]. The dislocation of the electrons is not uniform and could not be described with a dipole model. The dipolar plasmonic contributions are displayed at higher wavelengths; then, the quadrupolar and, in general, multipolar ones are shifted to shorter wavelengths.

## 7. Applications

### 7.1. Hyperthermia and Photothermal Therapy

**Figure 8:**
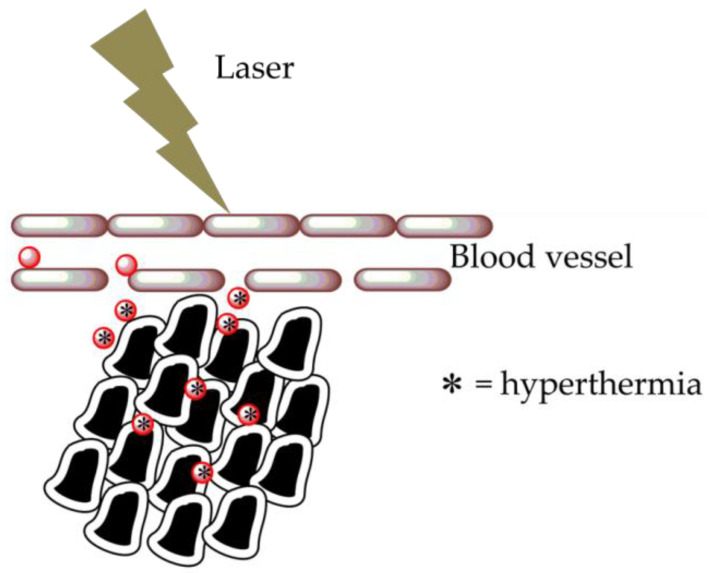
Hyperthermia cancer treatment using AuNPs. Nanoparticles carry a specific binder of the tumor, interacting with abnormal cells due to the implemented permeability of the vessels surrounding the cancer cells. Laser illumination of the AuNPs generates heat production.

Specific optical properties of AuNPs make them powerful nanometric thermal sources, thanks to an intrinsic energy exchange brought to light–heat conversion, generally called the thermo-optical response of nanomaterials. This response is mainly controlled by the behavior of electrons. In stationary conditions, a temperature variation provokes a modification of the material optical index and properties. The thermo-optical response of AuNPs is greatly influenced by the surface electromagnetic field improvement at the SPR [183]. AuNPs can achieve energy by absorbing photons, when exposed to visible incident light, through electron transitions. The relaxation process results in an individual electron–phonon collision and, then, a subsequent energy transfer from electrons to lattice vibrations. The SPR input is a powerful and rapid approach of absorption energy by light beam excitation and the conversion of this energy into heat on a nanoscale. When AuNPs are irradiated with an NIR light beam, the surface electrons are excited and thus they resonate. During electron relaxation, they radiate energy in a nonradiative way, and the surrounding temperature increases. This rise in temperature depends on the AuNP shape and concentration, as well as on their incubation time with tissues and the laser exposure time [184]. The typical absorption spectrum of AuNPs is related to their shape, and the wavelength range is usually between 650 and 900 nm, where the absorption due to tissue is minimal [185]. Gold nanorods have an enhanced length/width ratio, and the absorptive peak of their longitudinal SPR band shifts within the visible and NIR spectrums [186]. Heating could be to release drugs straight into a specific site. Moreover, the AuNP photothermal effect may be used to carry drugs across cell membranes, damage DNA, and produce oxygen-free radicals. Hyperthermia induces the localization of drugs inside a tumor cell by increasing the local blood flow. Furthermore, this condition works at the cellular level by enhancing the permeability and allowing a higher intracellular chemotherapy amount. Photothermal therapy uses NIR light absorption to cause thermal damage [185] by inducing the mechanisms of cellular damage that destroy cancer tissue [86], such as protein denaturation or tissue carbonization. Hyperthermia is based on heating an organ at temperatures between 41 and 45 °C; this therapy can also improve chemotherapy, laser-induced tumor damage [187] and also enhances the photodynamic (PDT) effect [188]. Hyperthermia is an interesting treatment with a lower side-effect profile than conventional cancer therapies (Figure 8). Customized therapy is based on “activated therapy”; in particular, enzyme-cleavable prodrugs are employed [189]. The activated prodrugs release the precursor drug after interacting with a specific biomarker within the cell [190]. Nanotechnology has supported the development of drug delivery systems employed in different clinical applications [191]. Even though there have been different drug delivery nanoparticle- or molecular-based systems all over the scientific bibliography, very few of them have been approved by the Medicines and Healthcare products Regulatory Agency (MHRA), the European Medicines Agency (EMA), or the US FDA, demonstrating difficulty in the clinical application of these nanosystems [192]. For instance, paclitaxel (Abraxane^®^, Abraxis BioScience Inc., Los Angeles, CA, USA), a 130-nm albumin particle, has been authorized by the US Food And Drug Administration (US FDA) for metastatic breast cancer [193]. Doxorubicin (another Doxil) is another example of an FDA-approved nanoparticle-based drug, which has been validated in metastatic ovarian cancer and AIDS-related Kaposi’s sarcoma therapies. A significant challenge for the implementation of the photothermal therapy effect is the homogeneous distribution of the temperature all over the tissue [194]. Methods that use temperatures above 45 °C to induce irreversible cell damage are related to thermal ablation techniques such as radiofrequency or microwave ablation. This causes a distinct area of cellular apoptosis surrounded by regions receiving less intense hyperthermia. Tumor cells seem to be more sensitive to heat-induced damage than healthy cells. In vivo tests show that tissue depths of approximately 1 cm could be irradiated with NIR light using untargeted AuNPs [195] without visible damage. In particular, the depth of penetration and the selectivity of photothermal therapy are some of the most important challenges for its employment in clinical tests, where tumor tissues may be 5–10 cm deeper. This phenomenon describes the recent research works and applications of AuNPs and their photothermal properties. 

### 7.2. AuNP for Health Applications

The combination of nanoscience and biotechnology has spawned nanobiotechnology; this research area offers a huge opportunity to advance in medical and health treatment, diagnostics, and therapeutics [44]. Among all noble metals, AuNP is the most largely studied thanks to its well-known synthesis procedures and safety profile. AuNP systems are considered a useful tool both in diagnosis and therapy (Theranostics) due to their singular properties, such as penetration and traceability within the body [196]. 

#### 7.2.1. Biodistribution and Cytotoxicity of AuNP

AuNP biodistribution and toxicity are essentially associated to the way they are introduced into the human body—namely, orally, intravenously, or directly into the target cell. Nanomaterial compositions and sizes are important parameter that regulate the cellular uptake mechanisms, the intracellular localization of AuNP, and their chemical interaction with cells [197]. A study on the influence of the nanoparticle size was carried out, considering the gastrointestinal absorption and the subsequent distribution of AuNPs in the tissue/organ. The latter were administered orally, in in vivo models, and different sizes of nanoparticles were investigated (58, 28, 10, and 4 nm) [198]. The presence of AuNPs in biological samples was qualitatively and quantitatively measured by the TEM analysis. The smallest (4 nm) nanoparticles were found in the kidneys, liver, lungs, spleen, and brain, while the largest (58 nm) AuNPs were mostly detected in the gastrointestinal tract. Through these distribution studies of AuNPs in tissues and organs, the paracellular mechanism suggested that heating could be used to release drugs straight into a specific site without being subjected to organized intracellular destructive processes, such as enzymatic degradation addressed to conjugated proteins or molecular species [199]. Oral and intravenous administrations are based on passive targeting, which, thanks to a greater permeation and retention effect, induces the accumulation of AuNP preferentially in the tumor site [200]. Several studies have shown that the reticuloendothelial system (RES) is the main route of elimination of AuNPs, occurring via macrophages in the liver and spleen. Therefore, the lower the interaction between AuNPs and RES, the higher the blood circulation time, with a consequent increase in intra-tumoral penetration [201]. Another in vivo study on the tissue distribution of different-sized AuNPs administered intravenously tested the presence of gold in different organs and tissues 24 h after injection. It was found that 70–80% of the total injected dose was present in the blood and the liver, regardless of the size of the AuNP [202]. 

Intra-tumoral administration is a direct method of introducing AuNPs directly into the tumor site [203]. A research study conducted ex vivo on a human eye affected by choroidal melanoma demonstrated the correct distribution of AuNPs within the tumor tissue; on the contrary, no nanoparticles were detected in the extra-tumoral areas [85]. Although this injection technique is able to provide a higher concentration of intra-tumoral AuNPs, resulting in a lower dose to administer, it may be difficult to treat tumors that are not accessible by direct injection [204].

The intracellular responses, the biodistribution, and the cytotoxicity of the nanoparticles depend on several factors, such as the size and shape, surface conjugations, the target cell type, and administration methods [205]. The cytotoxicity data are important to predict the AuNP biocompatibility [206]. Several studies have suggested the dependence of AuNP cytotoxicity on the doses, stabilizing agents employed [207], and target cell type [208]. Nonmalignant cells have been shown to be more sensitive to nanoparticles than cancer cells [209]. Even if a great number of research has shown low AuNP cytotoxicity [210], the wide available literature includes contradictory data because of the diverse cell lines, cell viability assays, the chemical routes employed for AuNP synthesis [211], and the absence of standard safety protocols. Several research studies have been conducted focusing on the relationship between the AuNP properties and cell death mechanisms for different types of tumor cells, and the cellular mechanisms studied are apoptosis, necrosis, and autophagy [212]. It was shown that smaller AuNPs tended to induce more necrosis, and hexagonal ones and nanorods causes more apoptosis, while AuNPs with hydrophobic functions induced greater apoptosis and autophagy than hydrophilic ones.

Another research study summarized the mechanism, the efficacy, and the toxicity of photothermal therapy by using AuNPs of different shapes and sizes [213]. The results showed that smaller AuNP sizes (≤20 nm) have longer blood retention and generate higher heat than larger nanoparticles, which showed a lower toxicity. Moreover, these particles are highly dependent on AuNP surface coating and cellular uptake behavior and cytotoxicity. In the previous paragraphs, different methods of AuNP synthesis were discussed, and in many of these, the surfactants are used as capping agents due to their cytotoxicity and the consequent limited use in clinical applications. To overcome this issue, a surface modification strategy using polymers was implemented and designed [214]. Numerous studies and research have been carried out to develop new AuNP nanosystems for anticancer therapies with potential clinical applications. In fact, the actual chemical use of these nanosystems is not yet applicable due to a series of problems associated with the targeted release of NPs at the tumor site, their biodistribution, and their intrinsic toxicity [215]. The latter is also related to the surface charge and size of AuNPs, with their consequent grouping, and their accumulation in particular biological sites [216]. A research work showed that AuNPs are more likely to accumulate in the liver and spleen, while they have not been detected in the heart, brain, kidneys, lungs, adrenal glands, or mesenteric lymph nodes [117]. However, experimental data are not sufficient to estimate the long-term AuNP cytotoxicity, and further investigations over longer time intervals are necessary.

#### 7.2.2. AuNPs as Delivery Carriers 

AuNPs have been used as an excellent system for the delivery of different types of drugs and biomolecules (DNA, RNA, and proteins) to the target sites [217]. The design of an efficient therapy, aimed to release the therapeutic agent, takes place by exploiting both the internal and the external conditions, such as the pH and the presence of oxidizing or reducing agents, and light. The main factor that significantly influences the drug release is the modification and functionalization of the nanocarrier surface [218]. The nonspecific targeting of AuNPs and their ability to stimulate the host’s immune system represent the main limitations in the use of these products as drug delivery systems. To tackle these problems, PEG modifications on the surfaces of AuNPs were carried out, with the aim of protecting the surface and inactivating them. This allowed also to minimize AuNPs’ tendency to stimulate the immune system [219]. In fact, this approach inhibited the adhesion of AuNPs on certain receptors, consequently making them “invisible” to the immune system. However, the specific functionalization of the surface can cause undesired toxic effects. For an effective anticancer therapy, the superficial functionalizations of AuNPs should be customized according to the chemoresistance and the diversity of the genetic makeup of the tumor cells. The efficacy of the anticancer drug transport nanosystems can be implemented by functionalizing the surfaces of the nanoparticles with stromal antagonists. This involves further studying the development of the previously described techniques, particularly with regards to active targeting [220]. Several biomolecules such as oligonucleotides, proteins, and peptides have been tested for targeted delivery to target cells using AuNP as the nanocarrier. In this regard, gene therapy has been found to be a highly efficient method for treating genetically acquired diseases, but it has also shown safety problems due to the random immune response and cytotoxicity [219]. For this reason, nonviral gene delivery systems were considered. An effective drug delivery system should allow facilitated entry into the cell, the protection of the nucleic acid from degradation by the nucleases, and the subsequent release of the nucleic acid in a functional form within the nucleus and the therapeutic effects of releasing all types of oligonucleotides, such as single-stranded or double-stranded DNA, plasmids, and single-stranded RNA [221]. Nucleic acid strands can be chemically modified with thiol groups to bind them to AuNPs covalently. It has been shown that AuNPs possess a high-affinity constant for the nucleotide sequence, showing a 99% higher cell internalization without causing cytotoxic effects, being also resistant to enzymatic degradation. Nucleic acids of an anionic nature can interact electrostatically with cationic AuNPs; in particular, a system of functionalized AuNPs with amino acids was created for the release of DNA, whose gene expression was much more efficient than the covalent functionalization of AuNPs [222]. Some studies have shown how AuNPs can identify the surface of an ionic protein through a complementary electrostatic interaction, limiting its activity [223]. For example, AuNPs functionalized and stabilized with chitosan can transport and release insulin, with a decrease in blood glucose levels after 2 h of oral administration [224]. These studies showed how functionalization can improve the efficiency and specificity of the nanoparticles in the target organ/tissue, revealing their potential use in nanopharmacology and nanomedicine [225]. The release of large biomolecules targeted on specific cells requires a prior step of cellular internalization before their release. Therefore, various factors need to be taken into account before using them for the delivery of biomolecules, such as the sizes and shapes of AuNPs, their functionalization, and their biodistribution and retention. A parameter that affects a drug’s release at the target site is the pH [226]. In particular, tumor cells have pH values ranging from 5.7 to 7.8. This condition causes both the breakdown of the bond sensitive to acids and variations of the total charge due to protonation and morphological alterations of the transported biomolecules [227]. Glutathione-mediated drug release is an alternative nonenzymatic approach for activating prodrugs in the intracellular environment. The underlying mechanism is the osmotic one, which exploits the difference in the glutathione concentration in the intracellular (1–10 mM) and extracellular matrix (2 μM) [228]. These methods focus on the formation of a disulfide bridge between drugs and their carrier. Along with the potential effectiveness of this approach, the modification of the reactivity conditions of the disulfide bond are challenging, mainly because of the collateral exchange reactions in the presence of cysteines localized on the surfaces of the blood proteins. This, in fact, causes the formation of different protein derivatives—transporters with different bioaccumulation and pharmacokinetic profiles.

### 7.3. Diagnostics

There is little research aimed at the direct use of AuNPs for cancer diagnostics and therapy [220] and even fewer technologies based on gold nanoparticles approved by the FDA for diagnostic and therapeutic purposes in medicine [229]. One of the clinical studies conducted by Astra Zeneca in collaboration with Cytimmune is mainly focused on the use of AuNPs for tumor therapy. Aurimune (CYT-6091) was used as a vehicle to transport recombinant human tumor necrosis factor alpha (rhTNF) into tumors, which allowed chemotherapy to enter cancer cells, damaging them. Thanks to the ability of AuNPs to absorb NIR light, the interest in photothermal conversion, selective targeting of tumor cells, and in vivo biodistribution of AuNPs has been growing [203]. The absorption of light causes a localized increase in temperature, resulting in the thermal dissolution of solid tumors [230]. An imaging technology has recently been developed during the focal ablation of prostate tumor tissue through direct laser irradiation from nanoparticles. This is the only ultra-focal tumor ablation therapy designed to implement therapy efficacy with minimal side effects. A recent clinical study based on AuNPs aimed to evaluate the feasibility of a new method used in oncology for the identification of gastric diseases based on the analysis of breaths with an array of nanosensors. The latter may be able to provide a noninvasive screening tool that distinguishes tumors located in the gastrointestinal tract from related precancerous lesions [231] and provides a diagnosis of pulmonary arterial hypertension.

#### 7.3.1. Enhanced Permeability and Retention Effect (EPR) and Tumor Targeting

The enhanced permeability and retention (EPR) effect supports a clarification for the specific targeting of AuNPs in the tumor cells [232]. As a consequence of tumor physiology, AuNPs selectively accumulate inside solid tumor tissues that are made of leaky blood vessels, with junction gaps varying the dimension from 100 nm to 780 nm [233], instead of normal capillaries, which have about 20-nm pore diameters [234]. Different research works have demonstrated that AuNPs up to 100 nm in size can pass through the reticuloendothelial system (RES) to accumulate in tumor tissues and be retained inside [235]. This is a passive method to convey AuNPs into tumor cells in order to irradiate them by photothermal therapy. This approach may be suitable for tumors less than 3 cm in size [236], but the most considerable restrain is the extensive biological heterogeneity of tumors and, therefore, the bio-specificity deficiency. Tumor tissues characterized by a poor vascularization, like prostate or pancreatic cancer, may not accumulate AuNPs only via the EPR effect. To increase the AuNP concentration inside tumor cells, active targeting has consequently been investigated by bonding a targeting side that is overexpressed in cancer cells [237]. Two different targeting mechanisms are employed to promote tumor specificity. AuNPs conjugated to a specific receptor are delivered through the typical mechanism for that particular receptor [238]. The most serious disadvantage related to active targeting is the dimensions of AuNPs that inhibits their transport across bio-barriers [239]. 

#### 7.3.2. Application of AuNPs for Small Molecule Detection

AuNPs can be employed as the Solid-Phase Extraction (SPE) adsorbent [240] as efficient sensors [241] for metal cation enhancement and revelation. For instance, dithiocarbamate functionalized diethanolamine (DEA) was employed to alter the AuNP surface, improving its affinity. The DEA is a symmetric compound used to chelate cations [242]. The synthesized DEA@AuNPs exhibit an adequate selectivity towards lead ions, depending on the coordination of the N and O DEA atoms, with the Pb^2+^ cations building a framework. AuNPs have been also used for the detection of environmental pollutants, such as polycyclic aromatic hydrocarbons (PAHs), based on the powerful affinity adsorption among the AuNP unmodified surfaces and PAHs: their determination was conducted by means of laser-excited time-resolved Shpol’skii spectrometry [243]. Furthermore, sixteen PAHs have been analyzed by using GC-MS with the support of AuNP-based extraction [244]. Some studies have connected AuNP-based nanoextraction with mass spectrometric detection [245]. AuNPs have been investigated for application in laser desorption ionization (LDI) mass spectrometry due to the high surface area and laser light absorbance, easy sample preparation, and analytical procedures [246]. Surface-modified and non-surface-modified nanoparticles have been utilized for detecting small molecules and ions, such as Hg^2+^ cations [247], amino thiols [245], and mono- and disaccharides [246]. The amount of detection was determined by a TOF MS analysis [247]. The results showed that unmodified AuNPs exhibit a stronger trapping efficiency for neutral carbohydrates and higher ionization efficiencies compared to capped AuNPs. Thanks to the great affinity of AuNPs towards thiol functional groups, AuNPs were employed for aminothiol compound extraction. AuNP detection was achieved by different methods, such as fluorescence detection [248] and capillary electrophoresis [249]. Hybrid materials of AuNPs with other materials have increasingly gained attention, especially for sensing with target specificity. For instance, a composite realized by mixing hybrid AuNPs and reduced graphene oxide has been tested as an adsorbing agent for the purification of mycotoxins and their HPLC-MS identification [250]. Polydopamine-stabilized magnetic AuNPs [133] have been synthesized for the detection of steroid hormones in milk, urine, and water samples. AuNPs were dispersed in an ionic solution of imidazolium functional group compounds prepared with the addition of pyridoxine (vitamin B6) and folic acid (vitamin B9) from the biological samples. The results of the HPLC-UV analysis showed a high selectivity, good extraction, and limit of detection [251]. The amphiphilic nature of the ionic solution enhanced the stability of the colloidal-modified AuNPs [252]. 

#### 7.3.3. Application of AuNPs for Detection of Biological Molecules

The most used device, developed for the detection of biological molecules such as proteins, hormones, and pesticides, is based on a polymeric membrane sheet where the sample is analyzed through lateral diffusion. The AuNP surface is modified with specific antibodies for selective detection exploiting an immune reaction. Once the target molecules sample is poured on the polymeric membrane sheet, the antibodies move together with the mobile phase, binding their corresponding antigen. This bond inhibits antibodies to bind the antigen, linked on a test line membrane, covalently. If the sample solution does not contain antigen molecules, the antibody sites are free to bind with the test line membrane, and the consequent accumulation of AuNPs causes the test line color to change; an increase in the amount of antigens corresponds to a decrease in the amount of antibodies bonded to the test line, thus resulting in the color fading. This method is employed to detect and characterize compounds such as hormones, pesticides, or drugs [253]. The sensitivity of the test is improved by adjusting the number of antigens linked to the AuNPs [254]. For the quantification and revelation of large proteins, a direct method is used: two different antibodies, both having a high affinity to the protein, are respectively bound to AuNPs and the line test. The target molecules bind to both antibodies, inducing the development of a colored line, whereas, in the absence of a protein, there is no binding, and no color change is observed [255]. The principle described for protein quantification is also used to develop devices for the rapid detection of polynucleotides [256]. The sensitivity of these devices may be improved by fixing an enzyme to the AuNP surfaces, and they can have different applications, as they are easy to use, compact, portable, and cost-effective.

### 7.4. Imaging

Several imaging techniques exploit the surface plasmon resonance effect characteristic of AuNP. Larger nanoparticles (400 nm) can be detected using an optical microscope in the phase contrast mode, which involves only scattered light in dark-field microscopy. Small AuNPs can only absorb light, causing the local heating of the environment, which can be detected by photothermal imaging, by fluorescence microcopy, which allows for single particle level detection, by multiphoton SPR microscopy, etc. Immunostaining is a TEM imaging technique based on AuNP conjugated with antibodies that bind fixed and permeabilized cell antigens [201]. The field of the research and development of innovative and highly efficient AuNP-based contrast agents for magnetic resonance imaging (MRI) is rapidly growing. The sensitivity of MRIs can be optimized by using AuNP as a carrier of gadolinium chelate models, currently utilized in the clinical diagnosis field. The core–shell particles of magnetite/AuNP employed in imaging have been synthesized thanks to the magnetic features of iron oxide (Fe_3_O_4_) and the optical properties of AuNP [257]. AuNP-assisted MRI could also be potentially used as a probe sensitive towards different types of proteins. Among the spectroscopic methods that characterize the electromagnetic field resulting from the plasmon resonance of AuNP surfaces, surface-enhanced Raman scattering (SERS) is the preferred one, since it allows a net enhancement of the signal and a limit of detection at the single-molecule level. The Raman effect in molecules far from the surface of an AuNP is weak, since the visible light not absorbed by these molecules is not dispersed in an anelastic way [258]. The intensity of the Raman signals on the AuNP surface is very high, because it is directly proportional to the fourth power of the local electric field, implemented thanks to the surface plasmon resonance and the charge transfer between the AuNP metal surface and the adsorbed molecules. The interference of the molecules that contribute to adsorption can prevent the detection of target molecules. The plasmon band moves from the visible region for spherical AuNPs to the NIR by changing the size, shape, and level of aggregation [259].

### 7.5. Application of AuNPs for the Biomarker Analysis

Biomarkers are exceptional and valuable indicators of a specific disorder. However, their analysis requires efficient sample preparation for a good selective extraction that needs to be suitable for the sensitive detection techniques [260]. Oxidized phospholipids are used as biomarkers for cardiovascular diseases, but an accurate analysis is not yet available. Haller et al. studied an AuNP nanoextraction method for trapping oxidized phospholipids through chemical identification by a bifunctional compound containing a hydrazide group for trapping phospholipid carbonyl groups and a thiol functional group for the selective bonding of AuNP derivatives. After the transamination of hydroxylamine, the oxime derivative of carbonylated phospholipids was analyzed by HPLC-ESI-MS/MS [261]. In this case study, different derivatization and releasing agents, used in different concentrations, were explored to develop an optimized sample preparation process for achieving strong selective enrichment and sensitive revelation. The correlation of the AuNP derivatives with the MALDI-TOF-MS analysis has recently shown significant improvement in sample homogeneity, decreasing the sample preparation time and removing the matrix ion interferences [262]. For instance, AuNPs functionalized with an aminooxy group were used for the chemical enrichment of glycosphingolipids (GSLs) in the living cell surface, and their identification was carried out by SPR and the MALDI-TOF-MS analysis. The AuNP trapping was found to be dependent of the ozonolysis reaction, which led to the formation of an oxime. Laser irradiation induced the oxime bond break and imino alcohol ion release for the MS analysis. Sudhir et al. reported the biomarker analysis for peptide and protein detection [263]. The results showed that the hydrophilic peptide methionine–encephalin and leucine–encephalin extractions were dependent on the AuNP surface charge and the target peptides’ isoelectric points (pI). The maximum extraction efficiencies were yielded above the peptides pI thanks to good ion pairing conditions at the considered pH value. Furthermore, unmodified AuNPs were also employed. For instance, Faccenda et al. studied unmodified AuNPs for the isolation of peptides containing a thiol group from the proteolysis of S-nitrosated proteins. The detection and characterization of the S-nitrosylation sites in the protein were carried out by MALDI-TOF [129]. In another study, bi-functionalized AuNPs were put together with a multivalent carbohydrate and a photoreactive site for the affinity extraction of carbohydrate-binding proteins, which were analyzed by the MALDI-TOF-MS analysis and fluorescence imaging after release by adding 2-mercaptoethanol [264]. This method allowed the simultaneous purification and characterization of carbohydrate-binding proteins. Combining functional groups could facilitate sample preparation, which is important for the analysis of low-abundance biomolecules, without interference and improve the sensitivity. For example, AuNP was functionalized with anti-insulin for trapping insulin in biofluid samples [265]. This characterization is very important, because it is one of the most meaningful post-translational protein modifications involved in different biological processes [266]. The high surface-to-volume ratio of AuNPs contributes to modifying the surface with different ligands to achieve the goal of affinity glycoprotein extraction and enrichment by suitable surface modification [267]. Tran et al. reported the synthesis of ultrasmall AuNPs, functionalized by hydrazide, which were used for the extraction and enrichment of *N*-glycosylated peptides. In this procedure, the AuNPs were modified with glutathione, and afterwards, the carboxylic acid groups were derivatives with hydrazine to obtain AuNPs@hydrazide. The extraction and enrichment procedure of the N-glycosylated peptides was realized after the reaction of aldehyde groups of carbohydrates with hydrazine groups on AuNP surfaces. The analysis was performed by HPLC and QTOF mass spectrometer [162]. Likewise, AuNPs functionalized with boronic acid exhibited a specific recognition of the glycan compounds, depending on the reversible covalent bonds between the acid and *cis*-diol groups. These AuNPs showed significant selectivity for the glycopeptides [268]. The AuNPs were also immobilized on monoliths to achieve enhanced surface reactive sites [267]. For instance, AuNPs grafted on poly(glycidylmethacrylate-co-poly (ethylene glycol) diacrylate) monoliths were functionalized with cysteine. Since grafting implemented the reactive sites and enhanced the hydrophilicity, this system was used for the efficient and selective enrichment of glycopeptides by hydrophilic interaction chromatography. The glycopeptides or deglycosylated peptides were analyzed by MALDI-TOF-MS and Nano RPLC-ESI-MS/MS [269]. AuNPs hybridized with different nanomaterials could improve the excellent properties of both materials and increase the application sphere [123].

### 7.6. Application of AuNPs as Bio-Barcodes

**Figure 9:**
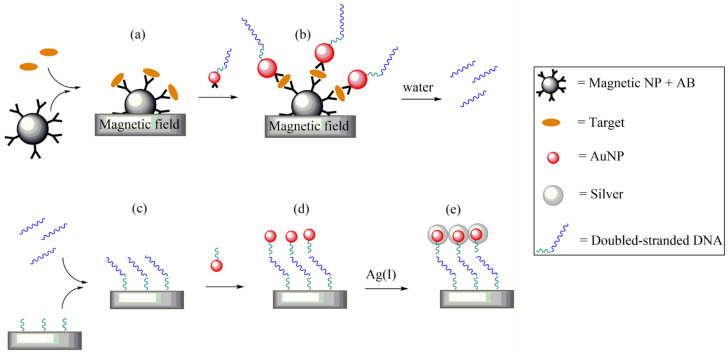
Functioning scheme of the AuNPs used in a bio-barcode. (**a**) Complex formed by a magnetic nanoparticle carrying specific antibodies and a target molecule, attracted on a magnetic substrate. (**b**) AuNPs bearing a double-stranded DNA and an antibody. (**c**) The double-stranded DNA washed away by water moves and interacts with a polynucleotide fixed to a chip. (**d**) Added AuNPs are functionalized by a complementary polynucleotide. (**e**) Finally, silver deposition allows the amplification of the detection signal.

Bio-barcodes are employed to quickly identify very low amounts of various proteins by using a series of reactions for the (i) specific detection, (ii) transcription, and (iii) amplification of the signal. The first reaction involves recognition of the target protein by binding specific antibodies to a magnetic substrate, even though the free proteins are washed away (Figure 9a). During the second reaction step, AuNPs carrying specific antibodies and oligonucleotides are both added; the antibodies are bound to allow the specific link of AuNPs to the proteins restrained by the magnetic substrate (Figure 9b). The transcription step involves the binding of AuNPs with an oligonucleotide linked to the chip, which is complementary to the sequence of antibodies fixed on the AuNP surfaces (Figure 9c). The last reaction consists of the reaction of Ag(I) with the AuNP surfaces in the presence of reducing agents, such as hydroquinone, resulting in the reduction and deposition of Ag(I) in the metallic nanoparticles (Figure 9d) for amplification (Figure 9e) of the signal related to the AuNPs [270]. This procedure promoted the amplification of the signal by increasing the AuNP sizes. To detect different proteins simultaneously, a two-dimensional array of oligonucleotides was used. Each oligonucleotide sequence corresponded to a specific antibody, so various changes of the protocols [271] approved the development of chips for bio-barcodes with a colorimetric reading or fluorescent biosensor [272].

## 8. Conclusions 

This review described in detail the studies and experimental results concerning AuNPs. In particular, the paper described different types of synthesis and functionalization methods, as well as various characterization techniques and possible biomedical applications, concerning AuNPs. The prospects for studying AuNPs are very promising; their synthesis can be done by different methods with no toxic effects, obtaining singular optical, physicochemical, and biological features. AuNPs present great potential in the modern biomedical field, and this review collected different synthesis methodologies that are used in the reduction of gold ions into metallic gold and the consequent functionalizations. In this paper, the most-used characterization methods to determine the sizes of AuNPs and their eventual functionalization are also illustrated. AuNPs also require a further stabilization analysis in biological fluids for in vitro and in vivo testing. The applications of functionalized AuNPs in medicine and biotechnology have highly developed recently. The latest research works, conducted under different experimental conditions and protocols, show conflicting results. The relative toxicity of AuNPs is still the subject of scientific research. Accurate therapeutic dosages, the delivery mechanism, and the absence of a toxicity database need to be discussed before the usage of nanocarriers in clinical trials. Antitumor-targeted drug delivery and biological marker systems are among the most important application fields. Metallic nanoparticles—in particular, AuNPs—have achieved great attention because of their size-dependent features and biological behavior, improved biocompatibility, stability, and oxidation resistance. AuNPs are appropriate systems for targeted and controlled drug delivery or even to enhance the external treatment potential. AuNPs are suitable agents for drug delivery systems because of the tenability of nanoparticle surfaces with various molecules, such as amino acids and peptides, oligonucleotides, antibodies, etc., to facilitate the loading of a drug. Drug delivery systems in which AuNPs behave as carriers represent an interesting application that requires more investigation to overcome the limitations and to improve the effectiveness and efficiency of drug release at the desired site. Gold has different chemical and physical properties, such as a high electronegativity, its tendency to link with other gold atoms, and its variable colors of dispersed colloids. These features are related to its electronic structure, and they explain the resulting suitability for its many applications. This review focused on the principles of thermo-optical properties emerging from plasmon resonance, as well as on some possible applications in biology, drug delivery, and therapy. 

## Data Availability

The data presented in this study are available on request from the corresponding author.
